# Evaluation of indigenous aromatic rice cultivars from sub-Himalayan Terai region of India for nutritional attributes and blast resistance

**DOI:** 10.1038/s41598-021-83921-7

**Published:** 2021-02-26

**Authors:** Debayan Mondal, Prudveesh Kantamraju, Susmita Jha, Gadge Sushant Sundarrao, Arpan Bhowmik, Hillol Chakdar, Somnath Mandal, Nandita Sahana, Bidhan Roy, Prateek Madhab Bhattacharya, Apurba Kr Chowdhury, Ashok Choudhury

**Affiliations:** 1 Department of Biochemistry, Uttar Banga Krishi Viswavidyalaya, Pundibari, Coochbehar, 736165 India; 2 Department of Plant Pathology, Uttar Banga Krishi Viswavidyalaya, Pundibari, Coochbehar, 736165 India; 3 Department of Seed Science and Technology, Uttar Banga Krishi Viswavidyalaya, Pundibari, Coochbehar, 736165 India; 4 ICAR-Indian Agricultural Statistics Research Institute, Pusa, New Delhi, 110012 India; 5 ICAR-National Bureau of Agriculturally Important Microorganisms, Mau Nath Bhanjan, UP, 275103 India; 6 Soil Microbiology Laboratory, Regional Research Station, Uttar Banga Krishi Viswavidyalaya, Pundibari, Coochbehar, 736165 India

**Keywords:** Biochemistry, Biotechnology, Molecular biology, Plant sciences

## Abstract

Indigenous folk rice cultivars often possess remarkable but unrevealed potential in terms of nutritional attributes and biotic stress tolerance. The unique cooking qualities and blissful aroma of many of these landraces make it an attractive low-cost alternative to high priced Basmati rice. Sub-Himalayan Terai region is bestowed with great agrobiodiversity in traditional heirloom rice cultivars. In the present study, ninety-nine folk rice cultivars from these regions were collected, purified and characterized for morphological and yield traits. Based on traditional importance and presence of aroma, thirty-five genotypes were selected and analyzed for genetic diversity using micro-satellite marker system. The genotypes were found to be genetically distinct and of high nutritive value. The resistant starch content, amylose content, glycemic index and antioxidant potential of these genotypes represented wide variability and ‘Kataribhog’, ‘Sadanunia’, ‘Chakhao’ etc. were identified as promising genotypes in terms of different nutritional attributes. These cultivars were screened further for resistance against blast disease in field trials and cultivars like ‘Sadanunia’, ‘T4M-3-5’, ‘Chakhao Sampark’ were found to be highly resistant to the blast disease whereas ‘Kalonunia’, ‘Gobindabhog’, ‘Konkanijoha’ were found to be highly susceptible. Principal Component analysis divided the genotypes in distinct groups for nutritional potential and blast tolerance. The resistant and susceptible genotypes were screened for the presence of the blast resistant *pi* genes and association analysis was performed with disease tolerance. Finally, a logistic model based on phenotypic traits for prediction of the blast susceptibility of the genotypes is proposed with more than 80% accuracy.

## Introduction

Rice (*Oryza sativa* L*.*) is one of the most popular staple foods consumed by more than half of the world’s population including Asians. Along with many south Asian countries, rice is widely consumed in India as cooked, puffed and pounded forms. Aromatic rice pertaining to a special group of rice is regarded highly due to presence of excellent aroma and superior grain quality. The Indian subcontinent is blessed with *nature’s gift* of Basmati rice popular among consumers as superior, scented, long slender grain rice and fetches premium price in the national and international markets. Along with the popular Basmati rice genotypes a hand full of traditional heirloom rice genotypes also possess excellent aroma, making many of these unrecognized landraces as an attractive low-cost alternative to high priced Basmati rice. West Bengal takes pioneer position in rice production in India and has considerably large diversity in cultivated folk rice genotypes. Two such cultivars of West Bengal, ‘Tulaipanji’ and ‘Gobindabhog’, have already been GI tagged and gained considerable attention in the international market for it’s uses in multinational cuisines and were featured in global sporting events^1^. Sub-Himalayan Terai region of West Bengal harbors considerably wide variability in rice cultivation and is identified as a hotspot of growing non-Basmati aromatic rice^2^. The rich agrarian heritage of the ancient tribe residents of this region, the ‘Rajbonshis’ has predominant role in conserving the rice agrobiodiversity of this region. The abundance of monsoon and large number of rivers in this region has facilitated the formation of Buri Balason rice bowl, Teesta-Dharala rice bowl, Kranti-Golabari rice bowl, Mekhliganj rice bowl etc. where aromatic landraces of rice, locally known as ‘bhogdhan’ are cultivated since time immemorial^3,4^. Besides aroma these local indigenous rice landraces, collectively regarded as the folk rice cultivars, offers genetic and nutritional diversity, sustainability, reduction in the carbon footprints and imports, tolerance to many diseases and pests due to the broadening of the gene pool, and adaptability to the local soil and climatic conditions^5^. The introduction of High-Yielding Varieties (HYV) in rice cultivation has led to gradual disappearance of folk rice cultivars from farmers field since green revolution^6–8^ which has resulted in survival of only a handful of these genotypes^9–13^. Although the replacement of these aromatic landraces with high- yielding modern varieties ensures good yield but enforces colossal threat to the rice agrobiodiversity resulting permanent damage to the rice ecosystem of different states of India^14^.

Aromatic landraces have comparatively low yield potentiality than HYVs, but offer considerable variation in agronomic, phenotypic, nutritional and disease tolerance traits. Inspite of their low yield potential, rice landraces have been proven valuable for resistance to rice blast disease in past^15,16^. The sub-Himalayan terai region being very hot and humid in nature, favors the spread of blast disease caused by fungus *Magnaporthe oryzae,* the most dreaded diseases in all rice growing regions of the world^17^. Profuse leaf blast is very common in this region causing significant yield loss of rice genotypes. Proper screening of the heirloom genotypes for the occurrence and severity of blast disease in this region has not been conducted till date and sources of resistance have not been investigated to explore the gene specificity. Blast pathogen is believed to follow the gene for gene hypothesis^17,18^. More than hundred blast-resistant (R) genes and around 350 QTLs has been reported conferring resistance to blast disease^15,19^ among which many has been cloned and biochemically characterized. There are absolutely no studies on the availability of blast-resistant (R) genes in these landraces. Mining of these R genes in these landraces is the need of the hour to combat region specific blast strain as the co-evolution of these genetically diverse landraces and the blast pathogen from time immemorial has enabled these genotypes with tolerance to the specific strain of the pathogen.

In the present study, we have performed genetic diversity analysis of locally grown scented aromatic landraces and determined the nutritive potential of these heirloom genotypes. The genotypes were also screened for resistance/tolerance to blast disease and suitable aromatic landraces were identified which can be used for future breeding programs. Allele mining for known source of resistance in these cultivars was performed. Our results portray the prospect of these scented landraces in terms of nutritional quality and blast disease resistance for the first time.

## Materials and methods

### Plant materials

The indigenous cultivars were collected from different parts of sub-Himalayan region of West Bengal and neighboring states. These genotypes are maintained in Uttar Banga Krishi Viswavidyalaya rice repository after purity breeding. For morphological characterization of the genotypes and screening for blast disease these genotypes were evaluated for two consecutive years (2018 and 2019 kharif season) in the field of Uttar Banga Krishi Viswavidyalaya Instructional Farm. IR64 was used as blast resistant check and ‘Gotra bidhan’ as local blast susceptible check.

### Field practices

The experimental field was divided into seventy-four plots of 5 m × 3 m size, having 1.5 ft spacing between plots and a 3 ft footway around the field. After seed viability test with 1% brine water, seed treatment was performed with tricyclazole (2 g per kg of seed) and sown in seedbeds. Twenty-five day old seedlings were transplanted in the main field with plant to plant spacing of 25 cm and line to line spacing of 50 cm. Fertilizer dose of N:P_2_O_5_:K_2_O_5_ at the rate of 70:50:40 kg/hectare (w:w) were applied. Half of ‘N’ has been applied as basal dose. Remaining half of ‘N’ was divided and applied after 30 days after transplanting (DAT) and before flowering stage. During the experiment, the daily weather reports having the parameters like rainfall (mm), maximum and minimum temperature (°C) and maximum and minimum relative humidity percentage were retrieved from the Integrated Agromet Advisory Services (Gramin Krishi Mousam Seva), UBKV, Coochbehar.

### Plant phenotypic parameters

All the phenotypic parameters were evaluated in two replications each year. Plant height, tiller number per plant, panicle number per plant, panicle length and leaf angles were measured after the panicle maturation whereas hundred seed weight, filled grain per panicle, grain length, grain width and yield parameters were taken after harvest and drying of the seed material. In each case mean value was calculated for both the years as an average of at least thirty plants taken from two replicative blocks. The upper leaf and lower leaf of every effective tiller was identified for measurement of leaf angle. First leaf after the flag leaf of the tiller was considered as upper leaf whereas the 3rd or 4th leaf from an effective tiller was considered as middle leaves. In each case effective tillers of at least 15 plants were considered and measured using a protractor at panicle maturation stage of the plant and average leaf angles were calculated.

### Disease scoring parameters

**Figure 1:**
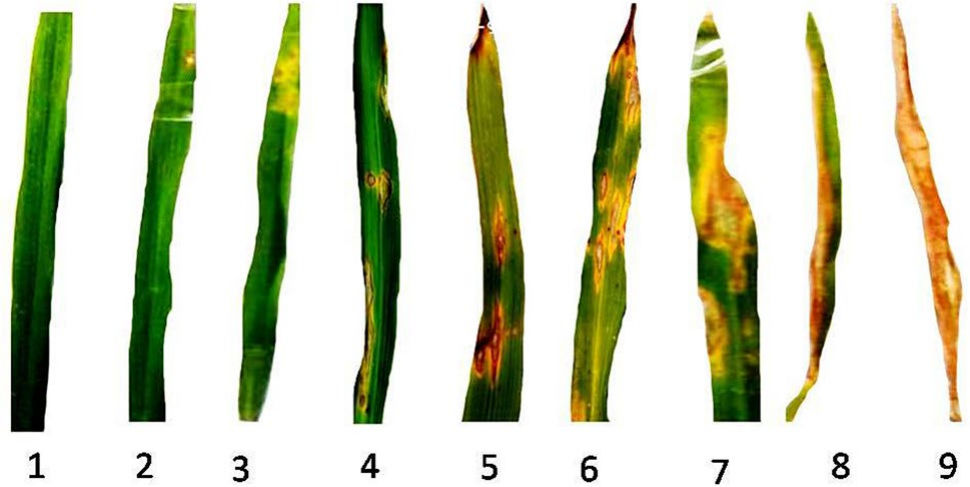
Pictorial representation of blast symptoms in the scale of 1 to 9 on rice leaves as evaluated for blast disease scoring.

The disease related parameters include blast disease scoring, lesion number in leaves, lesion size, lesion type and sporulation center. Disease scoring was done in 15 days interval starting from 3rd day of the month of August each year. Scoring of the symptoms was done following the standardized disease scoring scale defined by IRRI^20,21^. The minimum score of ‘0’ indicates of no disease and the maximum score of ‘9’ indicates severe disease symptoms of coalesced eye shaped spots on the leaf surface leading to complete drying of the leaves (Fig. 1). The Percentage Disease Index (PDI) values were calculated every 15-day interval for 2 months after transplanting to the main field from nursery and finally Area Under Disease Progress Curve (AUDPC) values were calculated from PDI values. The leaves with distinguished disease symptoms were considered for counting lesion number for each genotype in upper, middle and lower leaves. The typical spindle shaped brown lesions were counted in the leaves after 60 days of transplanting. Average lesion number was calculated from at least 50 such leaves for each case. The lesion area was calculated by measuring the length and breadth of the lesion using a millimeter scale. The lesion types were determined visually where the initial undefined yellow chlorotic lesions were marked as one where as fully matured brown necrotic lesion defined with white or gray center and brown lining in the periphery was defined as two. Average values for lesion type were calculated from at least 50 leaves for each genotype. Sporulation centre was considered based on the presence (one) and absence (zero) of the brown to black necrotic centers in the spindle shaped scars.

### In vitro disease progression assay

The infected leaf samples from the field were collected and the fungus was purified by single spore isolation method on Water Agar media, cultured on Potato Dextrose Agar media. The fungal DNA was isolated and sequenced. The ITS sequences were analyzed by NCBI-BLAST for confirmation. The leaf cuttings of different genotypes were infected with the confirmed culture of purified *Magnaporthe* for in-vitro disease progression assay. The progression of the disease was measured by appearance of symptoms in time lapse photography and subsequent microscopic studies.

### Nutritional parameters

**Figure 2:**
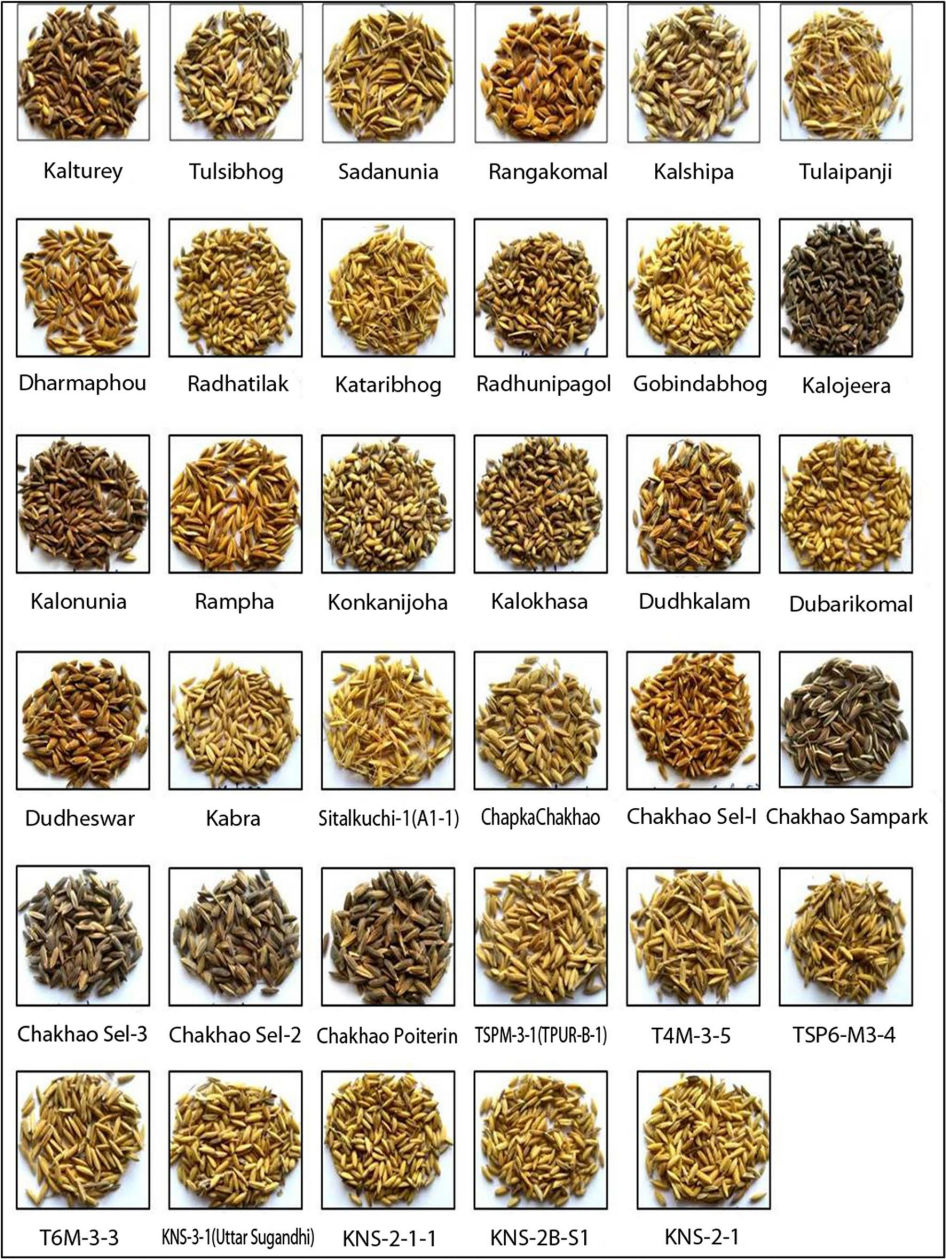
Paddy grain of the 35 aromatic landraces grown across sub-Himalayan Terai region of India. The low land region of north eastern India in the outer foothills of Himalaya and the north of the Indo-Gangetic plains is defined as the sub-Himalayan Terai region.

The grains of selected aromatic genotypes (Fig. 2) were harvested, cleaned, dehusked and powdered. These powdered samples were used for estimation of all biochemical parameters.

#### Carbohydrate parameters

The Total Soluble Sugars (TSS) and Starch (STA) content from rice powder was estimated by following Anthrone method^22^. The final absorbance was measured at 630 nm and the concentrations of Starch (STA) and Total Soluble Sugars (TSS) were calculated from a standard reference plot of glucose (10–100 µg). The Reducing and Non-Reducing sugars (RES and NRS) from rice powder were estimated by following DNS method^22^. The absorbance was measured at 510 nm. The concentration of Reducing and Non-Reducing Sugar (RES and NRS) were calculated from a standard reference plot of glucose (20–100 µg). The Amylose content (AMY) from rice powder was estimated by following iodine-colorimetric method^22^. The absorbance was taken at 590 nm. The concentration of amylose (%) was calculated from a standard reference plot of pure amylose from Potato (200–1000 µg). Amylopectin content (%) was determined by subtracting the amylose content from total starch content.

#### Protein content

The protein content from the rice powder was estimated by Bradford method^23^. The absorbance was recorded at 595 nm wavelength. The concentration of protein percentage was calculated from a reference plot of Bovine Serum Albumin (20–100 µg).

#### Resistant starch and glycemic index

The Resistant Starch (RS) of rice powder was estimated using Megazyme kit^24^. The absorbance was measured at 510 nm. Amyloglucosidase was used as a standard enzyme. Resistant Starch content was calculated using the formulae RS = ΔE × F/W × 9.27, where, ΔE = Absorbance, F/W = conversion of absorbance to micrograms (100 µg). The Glycemic Index (GI) of rice powder was estimated using in vitro method following Kumar et al*.*^24^. The absorbance was measured at 510 nm. Maltose (200 mg) was used as standard carbohydrate. Average values were used to plot curves followed by computing the area under the curve (AUC). The Hydrolysis index (HI) for each rice variety was calculated by dividing AUC of sample by that of maltose and expressed in percentage. The predicted Glycemic Index was calculated using the following formula (PGI) = 39.71 + (0.549 × HI).

#### Antioxidant activity

The antioxidant activity of the powdered rice was estimated by DPPH method^25^. A stock solution of DPPH (2, 2-diphenyl-1-picrylhydrazyl) was made by dissolving 24 mg in 100 ml methanol and working solution was prepared by diluting 10 ml of stock solution with 45 ml of methanol. The absorbance was recorded at 515 nm using UV–VIS spectrophotometer, along with control (Methanol—0.5 ml + DPPH—2.5 ml). The percentage of inhibition was expressed by putting the values into the formula:

$${\text{Percentage of Inhibition }}\left( \% \right) \, = \frac{{\left( {{\text{Absorbance of the control}} - {\text{ Absorbance of the test samples}}} \right) \, \times { 1}00}}{{\text{Absorbance of control}}}$$

The percentage of DPPH scavenging potential was plotted against the concentration of samples. The concentration of the sample necessary to decrease the DPPH concentration by 50% was obtained by interpolation from linear regression curve and denoted as IC_50_ value (μg/ml).

#### Aroma

The sensory test of rice aroma was performed by using 1.7% KOH solution to the rice powder following the method described by Hien et al*.*^26^. Based on aroma the genotypes were ranked as (1) mild, (2) medium, (3) strong. In a given day only 10 samples were evaluated as handling more may cause biasness.

### Genomic DNA isolation and genetic diversity analysis

The seeds of selected genotypes were geminated in water-soaked Petri plates under controlled condition. Genomic DNA was isolated from the young leaf tissue following the CTAB (cetyltrimethyl ammonium bromide) method with minor modifications. The quality of purified genomic DNA was estimated on 0.8% agarose gel electrophoresis. The DNA samples were later diluted with nuclease-free water to the concentration of 20 ng/μl and subjected to amplifications using SSR or ISSR markers^27,28^ and required PCR cocktail. The amplification of different alleles were scored as binary matrix where present or absent of an allele was denoted as 1and 0 respectively in an agarose gel (1.5–3%). Scoring was done on the basis of distinct, unambiguous and well resolved bands. Different parameters like heterozygosity index (H), polymorphic information content (PIC), resolving power, discriminating power etc. were calculated using iMec server (https://irscope.shinyapps.io/iMEC/)^29^. The UPGMA based genetic clustering was performed by NTSYS-PC version 1.80^30^.

### Allele mining for blast genes

Molecular screening, of the genotypes was performed for the presence of major blast resistant genes. The genotypes were differentiated on the basis of presence and/or absence of the above-mentioned blast resistant genes. PCR amplification was carried out in a 20 μl reaction volume containing 2 μl template DNA, (conc.) of Master Mix containing dNTP, MgCl_2_, Taq buffer and *Taq* DNA polymerase, forward and reverse primer and water. For scoring the marker genes the amplified PCR products were separated by electrophoresis in 1.5% agarose gels stained with ethidium bromide along with DNA ladder (NEB). All PCR reactions were carried out twice for reproducibility.

### Statistical analysis

#### Principal component analysis (PCA) and clustering

PCA have been carried out to identify the significant biochemical and disease related parameters. Based on PCA results, bi-plot analysis has been carried out to assess the impacts of different biochemical and phenotypic attributes respectively on different genotypes. The k-means non-hierarchical clustering algorithm was performed for grouping the rice genotypes based on different biochemical and phenotypic attributes. The number of clusters were determined using the gap statistic method. PCA and k-means non-hierarchical clustering techniques were carried out using R software version 3.5.1, Patched (2018-07-02 r74950) Platform: x86_64-w64-mingw32/x64 (64-bit)^31^. The correlation analysis between nutritional parameters and disease attributes were performed using SAS, version 9.3^32^.

#### Logistic regression analysis

Logistic regression modeling was carried out using IBM Statistical Package for the Social Sciences (SPSS trial version 20^33^) by considering only significant phenotypic characters except AUDPC as explanatory variable and presence or absence of blast disease as response variable where presence or absence of blast disease. If X_1_, X_2_,…X_p_ are p explanatory variables and if Y is the binary response variable taking value 0 and 1 for absence or presence of a particular attribute respectively, then the functional form of binary logistic regression equation is

$$\pi = P\left( {Y = 1} \right) = \frac{1}{{\left( {1 + e^{ - z} } \right)}} + \in$$

where π is the probability of an event occurrence under consideration and $z = \beta_{0} + \beta_{1} X_{1} + + \beta_{2} X_{2} + \ldots + \beta_{p} X_{p} .$ Here, $\in$ is the error term. The parameters of the equation are generally estimated through iterative maximum likelihood estimation procedure. The goodness of fit of the model was determined by Hosmer and Lemeshow goodness of fit test.

#### Kendall's tau-b correlation coefficient (τ_b_)

Kendall's tau-b correlation coefficient (τ_b_) was calculated using IBM-SPSS (SPSS trial version 20^33^) which is a nonparametric measure of association between two qualitative variables. Kendall's tau-b correlation coefficient (τ_b_) was calculated between presence of blast resistant genes and tolerance of the blast disease in different genotypes where tolerance or susceptibility of blast disease was addressed based on clustering of the genotypes and AUDPC values.

## Results and discussion

### Field attributes of indigenous farmer’s varieties collected from sub-Himalayan Terai region

A total of ninety-nine genotypes collected from different region of sub-Himalayan Terai region were subjected to purity breeding for at least five years and the pure lines were deposited to the Uttar Banga Krishi Viswavidyalaya (UBKV) rice repository. The performance of the genotypes for morphological and yield traits were assessed in the experimental plots. Majority of these genotypes are long duration, photosensitive, tall and have low yield potential (Table 1). The grains of these genotypes are predominantly long or short bold and very few genotypes have long to medium slender grains. Among the ninety-nine genotypes; twenty-seven were found to have mild to strong aroma among which only few genotypes exhibited very strong aroma. ‘Tulaipanji’, ‘Gobindabhog’, ‘Kalonunia’, ‘Radhunipagol’, ‘Konkanijoha’, ‘Chakhao’ etc. are traditionally known very important cultivars in northern Bengal as well as in different states of' north eastern India. Especially ‘Kalonunia’ and ‘Tulaipanji’ are two genotypes of great traditional value in Terai and Duars region and have excellent market potential. At UBKV eight photo-insensitive lines of these two cultivars have been developed keeping the fragrance intact using mutation breeding and selection programs^34^. Since the aroma is the most important criteria in terms of consumer preference of these genotypes, only thirty-five aromatic genotypes (twenty-seven farmers genotypes and eight UBKV developed lines from these genotypes) from total one hundred and seven genotypes (Supplementary information 1a, 1b, Table 1) were chosen for furthers studies. The detailed description of the selected genotypes along with their origin, ecology, place of collection and yield potential is given in Table 2.

**Table 1:** Mean performance of ninety-nine indigenous farmers’ varieties of rice (*Oryza sativa* L.) for yield and its attributes.

Sl. no.	Farmers’ varieties	X1	X2	X3	X4	X5	X6	X7	X8	X9	X10	Grain type	Aroma
1.	Ayangleima Phou	73.33	11.35	128.50	24.08	232.50	7.32	2.77	2.64	2.57	1.78	Long bold	No aroma
2.	Baigon Macchua	129.78	14.05	139.75	23.95	208.75	4.11	2.38	1.73	2.26	1.60	Short bold	No aroma
3.	Betho	137.95	22.20	138.00	23.78	140.25	5.64	2.51	2.25	2.34	2.01	Short bold	No aroma
4.	Beto	115.99	26.90	132.75	23.89	110.75	5.69	1.96	2.90	2.45	1.69	Short bold	No aroma
5.	Binni	127.85	20.00	125.25	23.54	118.05	7.19	2.21	3.25	2.69	1.56	Long slender	No aroma
6.	Birali	125.24	21.20	131.75	26.74	135.20	5.77	2.19	2.63	2.05	1.17	Short bold	No aroma
7.	Birali-Selection	146.65	14.90	138.25	24.57	152.00	7.45	2.20	3.39	2.93	2.26	Long slender	No aroma
8.	Boichi	136.04	13.90	134.00	26.08	113.45	5.84	2.83	2.06	2.22	1.93	Short bold	No aroma
9.	Bonnidhan	119.45	16.55	135.75	24.95	101.80	6.23	2.51	2.48	2.91	2.65	Long bold	No aroma
10.	Chakhao Amubi	125.60	14.35	128.75	23.56	132.95	6.38	2.61	2.44	2.41	2.04	Long bold	No aroma
11.	Chakhao Angangbi	124.56	20.50	117.75	23.11	105.55	7.16	2.71	2.64	2.50	1.61	Long bold	No aroma
12.	Chakhao Poireiton	136.61	15.75	119.25	26.65	175.75	6.44	2.40	2.68	2.49	1.54	Long bold	Strong
13.	Chakhao Sampark	129.07	13.75	120.00	25.25	132.08	7.15	2.73	2.62	2.70	1.55	Long bold	Medium
14.	Chakhao selection-1	127.80	21.90	133.25	22.41	166.85	5.96	2.59	2.30	2.28	2.06	Short bold	Medium
15.	Chakhao selection-2	111.32	11.50	119.50	22.95	162.14	6.52	2.34	2.79	2.58	1.88	Long slender	Medium
16.	Chakhao selection-3	118.29	19.45	120.50	22.66	188.25	5.70	2.72	2.10	2.31	1.72	Short bold	Medium
17.	Chapka Chakhao	84.77	18.45	126.75	20.06	150.30	6.50	2.27	2.86	2.39	2.11	Long slender	Mild
18.	Dharamphou	96.68	13.60	129.50	24.71	213.00	6.79	2.43	2.79	3.89	2.86	Long slender	Medium
19.	Dhyapa	127.24	23.60	132.75	24.27	174.85	5.80	2.70	2.15	2.86	2.31	Short bold	No aroma
20.	Dubarikomal	141.73	19.10	124.75	26.17	134.20	6.15	2.63	2.34	2.76	2.30	Long bold	Medium
21.	Dudhkalam Motajosawa	126.96	20.35	140.25	25.06	129.30	6.29	2.37	2.65	2.80	2.71	Long bold	No aroma
22.	Dudhkalam	141.77	15.70	140.50	28.18	161.40	6.16	2.44	2.52	2.45	2.21	Long bold	Mild
23.	Dudhkalam-9	111.73	18.35	139.75	28.44	137.90	7.38	2.55	2.89	2.71	1.51	Long slender	No aroma
24.	Dudheswar	124.62	20.00	127.25	25.37	118.35	6.31	2.57	2.46	2.44	1.43	Long bold	Medium
25.	Dudheswar-AD	123.04	15.20	135.75	28.35	195.55	6.61	1.94	3.41	2.05	1.49	Long slender	No aroma
26.	Fudugey	139.22	29.85	130.00	25.64	98.65	6.08	2.22	2.74	2.52	1.64	Long bold	No aroma
27.	Gobindabhog	124.05	15.20	138.00	26.27	231.95	4.32	1.71	2.53	1.34	1.36	Short bold	Strong
28.	Jaldhyapa-2	132.45	22.55	138.00	25.70	149.45	5.93	2.75	2.16	2.63	1.81	Short bold	No aroma
29.	Jaldhyapa-3	127.93	22.85	140.25	24.71	102.80	6.04	2.70	2.24	5.47	3.77	Long bold	No aroma
30.	Jaldhyapa-AD	132.05	24.10	138.00	27.34	134.60	6.89	2.85	2.42	3.35	2.36	Long bold	No aroma
31.	Jasawa-AD	121.90	14.90	122.00	26.80	157.10	6.06	2.79	2.17	2.85	2.23	Long bold	No aroma
32.	Jashyoya	132.86	14.95	138.75	25.27	108.35	6.03	2.78	2.17	4.38	3.51	Long bold	No aroma
33.	Jhapaka	155.09	15.35	111.00	25.22	112.20	6.58	2.49	2.64	2.31	2.08	Long bold	No aroma
34.	Jonroi Buna	169.76	19.00	125.50	22.79	139.85	5.59	2.74	2.04	2.51	1.71	Short bold	No aroma
35.	Kabra	120.19	26.60	136.00	26.37	140.05	6.13	2.10	2.92	2.15	1.72	Long slender	Strong
36.	Kagey	147.70	23.75	125.75	32.31	200.15	4.66	2.87	1.62	2.08	1.72	Short bold	No aroma
37.	Kaike	110.85	20.95	137.00	24.86	112.50	6.00	2.64	2.27	2.77	1.88	Short bold	No aroma
38.	Kaloboichi	103.29	23.90	136.00	21.43	105.95	6.63	2.27	2.92	2.42	1.71	Long slender	No aroma
39.	Kalodhyapa	120.92	24.95	136.00	25.62	151.00	6.10	2.61	2.34	2.34	1.84	Long bold	No aroma
40.	Kalojeera	130.62	20.95	136.00	27.50	146.75	4.22	2.36	1.79	1.44	1.41	Shot bold	Strong
41.	Kalokhasa	133.22	21.00	128.50	28.07	167.75	4.23	2.90	1.46	1.05	0.99	Short bold	Mild
42.	Kalonunia	137.64	13.30	137.25	25.29	114.65	5.30	1.96	2.70	1.56	0.95	Medium slender	Strong
43.	Kalshipa	116.82	24.00	134.50	25.14	140.70	5.91	2.36	2.50	2.25	2.03	Short bold	Mild
44.	Kalturey	157.41	13.90	119.00	25.31	98.85	6.02	1.99	3.03	1.41	1.33	Long slender	Strong
45.	KashiyaBinni	130.33	21.04	133.20	25.19	133.52	6.45	2.57	2.51	2.61	1.94	Long bold	No aroma
46.	Kataribhog	133.40	21.50	129.75	26.75	134.20	5.76	1.96	2.94	1.79	1.97	Medium slender	Medium
47.	Kauka-Selection	128.26	21.00	136.25	23.56	115.30	5.02	2.54	1.98	2.68	2.24	Short bold	No aroma
48.	Khaiyamdhan	135.75	25.50	133.50	25.73	105.95	6.45	2.59	2.49	2.65	2.24	Long bold	No aroma
49.	Kharadhan	145.55	17.35	131.75	29.52	211.60	6.36	2.51	2.53	4.38	2.83	Long bold	No aroma
50.	KonkoniJoha	128.05	19.75	131.25	29.01	273.52	4.36	2.43	1.79	1.42	1.31	Short bold	Strong
51.	Ladu	137.59	20.35	128.00	25.93	148.55	5.11	2.69	1.90	2.43	1.77	Short bold	No aroma
52.	Maitee	150.95	14.05	116.50	30.24	163.05	4.45	2.66	1.67	2.10	1.36	Short bold	No aroma
53.	Malbati	147.27	18.80	120.75	27.22	167.10	6.14	1.60	3.84	2.03	1.41	Long bold	No aroma
54.	Malshira	118.55	21.20	131.00	26.64	137.80	6.12	2.22	2.76	2.20	2.59	Long slender	No aroma
55.	Mangamuthi	161.53	16.85	134.50	27.41	163.40	6.75	3.04	2.22	3.27	2.28	Long bold	No aroma
56.	Pahariboichi	117.58	23.55	131.75	24.87	158.40	5.90	2.55	2.31	2.04	2.04	Short bold	No aroma
57.	Pahariboichi-Selection	128.91	20.65	116.25	26.28	164.05	5.46	2.38	2.29	2.34	1.86	Short bold	No aroma
58.	PanikuthiShyamlal	134.96	21.95	140.75	26.55	149.15	6.09	2.31	2.64	3.34	2.00	Long bold	No aroma
59.	Phoolpakari	116.36	25.25	134.50	24.11	141.45	5.76	1.95	2.95	1.49	1.39	Medium slender	No aroma
60.	Phoolpakari-1	116.86	18.50	129.00	22.28	129.35	5.21	1.86	2.80	1.82	1.34	Medium slender	No aroma
61.	Phorenmubi	123.79	15.05	120.75	26.41	140.25	7.07	2.14	3.30	2.64	1.61	Long slender	No aroma
62.	Radhatilak	127.65	14.95	129.75	24.70	241.75	4.96	1.94	2.56	1.50	1.28	Short bold	medium
63.	Radhatilak-2	119.84	16.40	136.50	24.31	161.50	4.34	1.93	2.25	1.12	1.02	Short bold	No aroma
64.	Radhunipagol	144.08	21.75	133.25	25.97	173.75	4.57	1.92	2.38	1.56	1.48	Short bold	Strong
65.	Rampha	139.84	16.45	132.75	25.35	122.65	6.05	1.98	3.06	2.15	2.16	Long slender	Strong
66.	Rongakomal	134.55	20.75	129.50	24.54	156.75	5.11	2.82	1.81	2.58	2.05	Short slender	Medium
67.	Sada Mala	101.70	17.40	127.75	24.66	152.10	6.37	2.14	2.98	2.03	1.55	Long slender	No aroma
68.	Sadanunia	105.06	13.80	91.75	28.72	124.40	7.81	2.97	2.63	2.11	1.85	Extra-long slender	Medium
69.	Sadabhatkalo	131.59	21.35	138.50	24.91	187.80	6.72	2.52	2.67	2.58	2.20	Long bold	No aroma
70.	Satia	124.37	23.30	131.25	24.26	121.25	5.60	2.25	2.49	2.29	2.14	Short bold	No aroma
71.	Seshphal	83.14	14.95	95.75	24.09	166.70	5.30	1.88	2.82	1.88	1.89	Medium slender	No aroma
72.	Sitalkuchi-1 (A-1-1)	112.97	12.35	132.25	28.14	128.95	5.31	2.86	1.86	2.50	2.20	Short bold	Medium
73.	Sitalkuchi-2	122.04	13.85	136.75	23.90	166.50	5.80	2.60	2.23	2.63	2.12	Short bold	No aroma
74.	Sitalkuchi-3	128.22	23.20	130.50	24.68	147.02	6.13	2.91	2.11	2.97	2.84	Long bold	No aroma
75.	Sitalkuchi-5	105.79	20.75	130.50	27.00	110.90	5.58	2.45	2.28	2.19	1.94	Short bold	No aroma
76.	Sitalkuchi-6	122.95	19.50	136.50	25.78	107.17	6.49	2.36	2.75	2.38	1.61	Long bold	No aroma
77.	Tarai Research Society-1	122.80	21.00	137.00	25.35	151.35	5.70	2.62	2.18	2.41	1.35	Short bold	No aroma
78.	Tarai Research Society-2	112.75	23.65	126.50	23.71	98.50	6.31	2.39	2.64	2.33	1.59	Long bold	No aroma
79.	Tarai Research Society-3	117.18	15.95	136.50	24.13	267.08	4.18	2.36	1.77	2.07	1.96	Short bold	No aroma
80.	Tarai Research Society-4	123.01	14.25	134.00	25.09	135.40	5.21	1.87	2.79	1.76	1.60	Medium slender	No aroma
81.	Tarapakari	118.59	22.00	136.50	25.53	198.15	4.88	1.82	2.68	2.07	1.61	Medium slender	No aroma
82.	Tarapakari-Selection	131.62	21.90	132.50	27.46	177.70	4.70	2.47	1.90	2.29	2.22	Short bold	No aroma
83.	Thuri	114.73	16.60	141.00	21.76	282.10	6.19	2.33	2.66	2.43	2.06	Long bold	No aroma
84.	Tulaipanji	118.47	27.75	131.50	24.66	97.85	6.18	1.84	3.36	1.44	0.83	Long slender	Strong
85.	Tulsibhog	136.78	25.10	131.50	24.11	123.85	5.44	2.01	2.71	1.47	1.17	Short bold	Strong
86.	Tulsimukul	141.12	16.30	138.00	30.61	170.30	4.45	2.19	2.03	1.57	1.68	Short bold	No aroma
87.	Uttar Banga Loca-3	127.68	21.00	139.75	27.23	178.90	5.71	2.43	2.35	2.75	2.16	Short bold	No aroma
88.	Uttar Banga Local-10	109.93	15.00	118.50	26.21	124.00	8.08	2.00	4.04	3.17	1.79	Extra-long slender	No aroma
89.	Uttar Banga Local-11	131.72	15.60	133.75	25.64	215.10	5.39	2.35	2.29	2.01	2.10	Short bold	No aroma
90.	Uttar Banga Local-13	135.50	19.75	133.75	25.40	176.50	4.36	2.07	2.11	1.56	1.80	Short bold	No aroma
91.	Uttar Banga Local-14	128.00	19.05	136.75	23.04	170.95	7.80	2.29	3.41	3.37	2.09	Extra-long slender	No aroma
92.	Uttar Banga Local-15	126.86	22.25	133.50	26.38	106.60	5.61	2.43	2.31	2.59	2.13	Short bold	No aroma
93.	Uttar Banga Local-17	131.27	21.55	133.00	29.74	206.25	6.21	2.03	3.06	2.58	1.69	Long slender	No aroma
94.	Uttar Banga Local-18	89.37	12.40	140.00	24.76	139.50	7.05	1.80	3.92	1.81	1.73	Long slender	No aroma
95.	Uttar Banga Local-2-AD	121.49	21.25	130.50	25.66	110.45	6.08	2.26	2.69	1.82	1.48	Long bold	No aroma
96.	Uttar Banga Local-3-1	140.96	20.65	136.00	26.31	98.85	5.61	2.74	2.05	2.22	2.03	Short bold	No aroma
97.	Uttar Banga Local-5	113.13	13.40	137.50	24.96	254.17	4.29	2.68	1.60	2.09	1.43	Short bold	No aroma
98.	Uttar Banga Local-6	115.74	20.25	136.25	25.87	108.45	6.33	2.55	2.48	2.45	1.46	Long bold	No aroma
99.	Uttar Banga Local-9	131.33	25.20	132.50	29.09	142.90	6.66	2.33	2.86	2.22	1.84	Long bold	No aroma
100.	KNS-2′-1	108.69	16.77	118.45	22.55	168.26	5.25	1.81	2.90	1.51	2.14	Medium slender	Strong
101.	KNS-3′-1 Uttar Sugandhi (IET 24616)	107.82	22.05	120.50	25.00	179.73	5.04	1.87	2.69	1.48	3.33	Medium slender	Strong
102.	KNS-2-1-1	116.51	18.10	119.35	18.80	153.47	5.30	1.83	2.89	1.53	1.94	Medium slender	Strong
103.	KNS-2B-S1	111.92	20.40	123.45	16.85	161.29	5.55	1.92	2.89	1.60	2.05	Medium slender	Strong
104.	T4M-3-5	85.80	17.15	120.00	21.00	112.00	6.24	2.19	2.85	1.73	3.06	Long slender	Strong
105.	TSP6-M3-4	81.83	21.73	122.00	23.00	107.40	6.31	2.35	2.80	1.76	3.22	Long slender	Strong
106.	TPUR-B-1 (IET 28104)	92.12	20.62	118.00	23.40	120.40	6.45	2.13	3.03	1.71	2.13	Long slender	Strong
107.	T6M-3–3	80.40	21.22	117.00	23.60	115.60	6.27	2.21	2.84	1.59	2.69	Long slender	Strong

*X1: *plant height (cm), *X2*: panicle no./plant, *X3: *days to 50% flowering, *X4: *panicle length (cm), *X5: *filled grain/panicle, *X6: *decorticated grain length (mm), *X7: *decorticated grain width (mm), *X8: *L:B ratio, *X9: *100-seed weight (g), *X10: *yield (t/ha).

**Table 2:** Description of the selected traditionally important aromatic cultivars along with photo insensitive line developed from these aromatic cultivars.

Sl no.	Name of the genotype	Description of the genotype	Origin	Ecology	Parentage	Place of collection/source of the seed
1.	Tulaipanji	Photo-period sensitive, long duration, medium tall, medium slender grain with long awn, lodging susceptible, low yield potential (1.5–2.0 t/ha)	Raiganj sub-division, Uttar dinajpur district, West Bengal	Medium or low land	Landrace	Uttar Dinajpur KVK, West Bengal
2.	Radhatilak	Photo-period sensitive, long duration, tall, short bold grain, lodging susceptible, low yield potential (3.0–3.5 t/ha)	Northern part of West Bengal	Medium or low land	Landrace	Tarai Research Society, Alipurduar, West Bengal
3.	Kalshipa	Photo-period sensitive, long duration, tall, bold grain, lodging susceptible, low yield potential (2.5–3.0 t/ha)	Northern part of West Bengal	Medium or low land	Landrace	Tarai Research Society, Alipurduar, West Bengal
4.	Rangakomal	Photo-period sensitive, long duration, tall, bold grain, brown-red grain husk, lodging susceptible, low yield potential (2.0–2.5 t/ha)	Lower-eastern part of Assam	Medium or low land	Landrace	ICAR-CPCRI- Kahikuchi, Kamrup, Assam
5.	Sadanunia	Photo-period sensitive, medium duration, medium tall, long slender grain with long awn, low yield potential (1.5–2.0 t/ha)	Northern part of West Bengal	Medium land	Landrace	Tarai Research Society, Alipurduar, West Bengal
6.	Tulsibhog	Photo-period sensitive, long duration, tall, medium slender grain, low yield potential (1.5–2.0 t/ha)	Northern part of West Bengal	Medium or low land	Landrace	Tarai Research Society, Alipurduar, West Bengal
7.	Kalturey	Photo-period sensitive, long duration, tall, highly lodging susceptible, medium slender grain, low yield potential (1.5–2.0 t/ha)	Darjeeling Hills	Hill slope	Landrace	Darjeeling KVK, West Bengal
8.	Kataribhog	Photo-period sensitive, long duration, tall, slender grain, low yield potential (1.5–2.0 t/ha)	Northern part of West Bengal	Medium or low land	Landrace	Tarai Research Society, Alipurduar, West Bengal
9.	Sitalkuchi-1(A1-1)	Photo-period sensitive, long duration, tall, slender grain, low yield potential (1.5–2.0 t/ha)	Sitalkuchi block, Cooch Behar district, Northern part of West Bengal	Medium or low land	Selection from Sitalkuchi-1	Sitalkuchi block, Cooch Behar district, West Bengal
10.	Dubarikomal	Photo-period sensitive, long duration, tall, short-bold grain, brown-red grain husk, lodging susceptible, low yield potential (2.0–2.5 t/ha)	Lower-eastern part of Assam	Medium or low land	Landrace	ICAR-CPCRI- Kahikuchi, Kamrup, Assam
11.	Dharmaphou	Photo-period sensitive, long duration, tall, bold grain, low yield potential (2.5–3.0 t/ha)	Lower-eastern part of Assam	Medium or low land	Landrace	ICAR-CPCRI- Kahikuchi, Kamrup, Assam
12.	Dudheswar	Photo-period sensitive, long duration, tall, short-bold grain, good eating quality, low yield potential (2.5–3.0 t/ha)	Northern part of West Bengal	Medium or low land	Landrace	Tarai Research Society, Alipurduar, West Bengal
13.	Dudhkalam	Photo-period sensitive, long duration, tall, short-bold grain, good eating quality, low yield potential (2.5–3.0 t/ha)	Northern part of West Bengal	Medium or low land	Landrace	Tarai Research Society, Alipurduar, West Bengal
14.	Rampha	Photo-period sensitive, long duration, tall, bold grain, low yield potential (2.0–2.5 t/ha)	Lower-eastern part of Assam	Medium or low land	Landrace	ICAR-CPCRI- Kahikuchi, Kamrup, Assam
15.	Gobindabhog	Photo-period sensitive, long duration, tall, short-bold grain, good eating quality, low yield potential (3.0–3.5 t/ha)	Lower western part of West Bengal (Burdhawan district)	Medium or low land	Landrace	BCKV, Mohanpur, West Bengal
16.	Konkanijoha	Photo-period sensitive, long duration, tall, short-bold grain with black husk, low yield potential (2.0–2.5 t/ha)	Lower-eastern part of Assam	Medium or low land	Landrace	ICAR-CPCRI- Kahikuchi, Kamrup, Assam
17.	Kalokhasa	Photo-period sensitive, long duration, tall, short-bold grain, good eating quality, low yield potential (3.0–3.5 t/ha)	Murshidabad district, West Bengal	Medium or low land	Landrace	Murshidabad district, West Bengal
18.	Kabra	Photo-period sensitive, long duration, tall, bold grain, low yield potential (2.0–2.5 t/ha)	Lower-eastern part of Assam	Medium or low land	Landrace	ICAR-CPCRI- Kahikuchi, Kamrup, Assam
19.	Radhunipagol	Photo-period sensitive, long duration, tall, lodging susceptible, short-bold grain, low yield potential (2.0–2.5 t/ha)	Lower western part of West Bengal	Medium or low land	Landrace	BCKV, Mohanpur, West Bengal
20.	Kalojeera	Photo-period sensitive, long duration, tall, lodging susceptible, short-bold grain with black husk, low yield potential (1.5–2.0 t/ha)	Lower western part of West Bengal	Medium or low land	Landrace	BCKV, Mohanpur, West Bengal
21.	Kalonunia	Photo-period sensitive, long duration, tall, lodging susceptible, medium slender grain, low yield potential (1.5–2.0 t/ha)	Northern part of West Bengal	Medium or low land	Landrace	Rice Repository, UBKV, Cooch Behar
22.	Chakhao Sel-I	Photo-period sensitive, long duration, medium tall, bold grain with black kernel, low yield potential (1.5–2.0 t/ha)	Selection from Chakhao	Medium or low land	Landrace	Rice Repository, UBKV, Cooch Behar
23.	Chakhao Poiterin	Photo-period sensitive, long duration, medium tall, bold grain with black kernel, low yield potential (1.5–2.0 t/ha)	Manipur	Medium or low land	Landrace	Manipur
24.	Chapka Chakhao	Photo-period sensitive, long duration, medium tall, bold grain with black kernel, low yield potential (1.5–2.0 t/ha)	Manipur	Medium or low land	Landrace	Manipur
25.	Chakhao Sel-2	Photo-period sensitive, long duration, medium tall, bold grain with black kernel, low yield potential (1.5–2.0 t/ha)	Selection from Chakhao	Medium or low land	Landrace	Rice Repository, UBKV, Cooch Behar
26.	Chakhao Sel-3	Photo-period sensitive, long duration, medium tall, bold grain with black kernel, low yield potential (1.5–2.0 t/ha)	Selection from Chakhao	Medium or low land	Landrace	Rice Repository, UBKV, Cooch Behar
27.	Chakhao Sampark	Photo-period sensitive, long duration, medium tall, bold grain with black kernel, low yield potential (1.5–2.0 t/ha)	Manipur	Medium or low land	Landrace	Manipur
28.	KNS-2-1	Photo-period sensitive, long duration, tall, lodging susceptible, medium slender grain, low yield potential (1.5–2.0 t/ha)	Selection from KaloNunia	Medium or low land	Landrace	Rice Repository, UBKV, Cooch Behar
29.	KNS-3-1 (Uttar Sugandhi) (IET 24616)	Photo-period sensitive, long duration, tall, lodging susceptible, medium slender grain, low yield potential (2.0–2.5 t/ha)	Selection from KaloNunia	Medium or low land	Landrace	Rice Repository, UBKV, Cooch Behar
30.	KNS-2-1-1	Photo-period sensitive, long duration, tall, lodging susceptible, medium slender grain, low yield potential (2.0–2.5 t/ha)	Selection from KaloNunia	Medium or low land	Landrace	Rice Repository, UBKV, Cooch Behar
31.	KNS-2B-S1	Photo-period sensitive, long duration, tall, lodging susceptible, medium slender grain, low yield potential (2.0–2.5 t/ha)	Selection from KaloNunia	Medium or low land	Landrace	Rice Repository, UBKV, Cooch Behar
32.	T4M-3-5	Photo-period insensitive, medium duration, semi-dwarf, lodging tolerant, medium slender grain with long awn, medium yield potential (3.5–4.0 t/ha)	Tulaipanji mutant	Medium and upland	Mutant of Tulaipanji	Rice Repository, UBKV, Cooch Behar
33.	TSP6-M3-4	Photo-period insensitive, medium duration, semi-dwarf, lodging tolerant, medium slender grain with long awn, medium yield potential (3.5–4.0 t/ha)	Tulaipanji mutant	Medium and upland	Mutant of Tulaipanji	Rice Repository, UBKV, Cooch Behar
34.	TSPM-3-1 (TPUR-B-1-IET 28104)	Photo-period insensitive, medium duration, semi-dwarf, lodging tolerant, medium slender grain with long awn, medium yield potential (3.5–4.0 t/ha)	Tulaipanji mutant	Medium and upland	Mutant of Tulaipanji	Rice Repository, UBKV, Cooch Behar
35.	T6M-3-3	Photo-period insensitive, medium duration, semi-dwarf, lodging tolerant, medium slender grain with long awn, medium yield potential (3.5–4.0 t/ha)	Tulaipanji mutant	Medium and upland	Mutant of Tulaipanji	Rice Repository, UBKV, Cooch Behar

### Genetic diversity of indigenous aromatic cultivars using microsatellite markers

**Figure 3:**
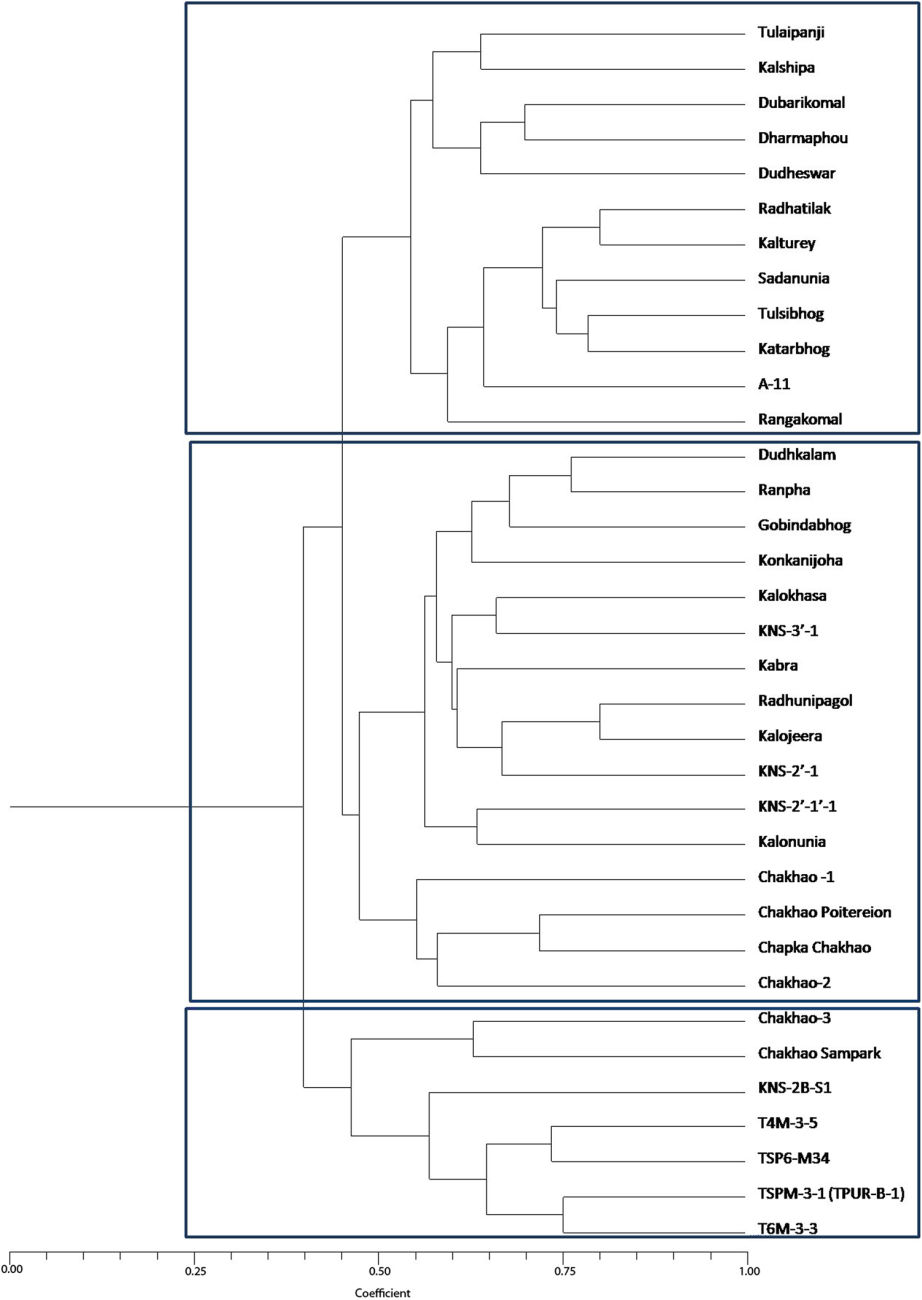
UPGMA based genetic clustering of thirty-five aromatic landraces determined by polymorphism obtained from SSR and ISSR markers using NTSYS-PC version 1.80^30^ (http://www.exetersoftware.com/cat/ntsyspc/ntsyspc.html).

A total of forty-two microsatellite markers (thirty-two SSR and ten ISSR) were used for evaluating genetic diversity of the selected aromatic genotypes (Table 3)^27,28^. A total of 45 alleles were detected using SSR markers and 30 alleles were detected using purine rich ISSR markers. Except RM469 all the SSR markers were found to be polymorphic in nature. Percentage polymorphism varied between 14.29% for RM460 to 97.14% for RM108 and RM434. Polymorphism Information Content (PIC) value varied from 0.052 (RM434) to 0.375(RM288) with highest Heterozygosity index (5.00) (Table 4). RM 1, RM23, RM 38, RM 250, RM 314, RM 3134, RM 3872, RM 6250 etc. exhibited good resolving and discriminatory power among the SSR markers. All the purine rich ISSR Markers exhibited very good discriminatory power and high PIC ranging from 0.295 to 0.374 and proved to be more effective for diversity analysis. Based on the microsatellite marker analysis genetic clustering was performed using NTSYS software (Fig. 3, Supplementary information 2a, 2b). Clustering using UPGMA method grouped the total thirty-five accessions into three distinct clusters. The distance coefficient of these clusters ranged from 0.45 to 0.85. Cluster I consisted of two subgroups, subgroup I has five genotypes i.e. ‘Tulaipanji’, ‘Kalshipa’, ‘Dubarikomal’, ‘Dharmaphou’ and ‘Dudheswar’, majority of these genotypes are photo sensitive, long duration, tall, lodging susceptible having low yield potential (2–2.5 t/ha). Seven genotypes, ‘Radhatilak’, ‘Kalturey’, ‘Sadanunia’, ‘Tulsibhog’, ‘Kataribhog’, ‘A-1-1’ and ‘Rangakomal’ constitute Sub group II. Majority of these genotypes exhibits very low yield potential (1.5–2 t/ha). The second cluster constitutes of sixteen genotypes amongst them four genotypes is black rice introduced from north eastern Manipur state and the remaining genotypes, ‘Kalonunia’, ‘Gobindabhog’, ‘Radhunipagol’, ‘Konkanijoha’, ‘Rampha’ etc. are known for their excellent aroma. The selections from ‘Kalonunia’ with strong aroma are found in this cluster. The third cluster is majorly comprised of comparatively high-yielding photo insensitive lines of ‘Tulaipanji’ and ‘Kalonunia’ along with two ‘Chakhao’ cultivars. Yield potential of this cluster varied from 3.5 to 4 t/ha.

**Table 3:** Detailed of the SSR and ISSR markers used in the study.

Primer ID	Chr no	Forward sequence	Reverse sequence	Monomorphic/polymorphic	Allele no	Amplicon size (bp)	Annealing temp (°C)	% Polymorphic
RM 1	1	GCGAAAACACAATGCAAAAA	GCGTTGGTTGGACCTGAC	Polymorphic	1	100	51	20.00
RM 23	1	CATTGGAGTGGAGGCTGG	GTCAGGCTTCTGCCATTCTC	Polymorphic	3	50–350	55	15.24
RM 38	8	ACGAGCTCTCGATCAGCCTA	TCGGTCTCCATGTCCCAC	Polymorphic	1	900	59	22.86
RM 108	9	TCTCTTGCGCGCACACTGGCAC	CGTGCACCACCACCACCACCAC	Polymorphic	1	80	66	97.14
RM 114	3	CAGGGACGAATCGTCGCCGGAG	TTGGCCCCCTTGAGGTTGTCGG	Polymorphic	2	200–450	66	80.00
RM 159	5	GGGGCACTGGCAAGGGTGAAGG	GCTTGTGCTTCTCTCTCTCTCTCTCTCTC	Polymorphic	4	150–650	55	89.29
RM 165	1	CCGAACGCCTAGAAGCGCGTCC	CGGCGAGGTTTGCTAATGGCGG	Polymorphic	2	200–300	58	34.29
RM 169	5	TGGCTGGCTCCGTGGGTAGCTG	TCCCGTTGCCGTTCATCCCTCC	Polymorphic	2	200–900	62	62.86
RM 172	7	TGCAGCTGCGCCACAGCCATAG	CAACCACGACACCGCCGTGTTG	Polymorphic	1	180	51	40.00
RM 195	8	AGAAAGAGAGGCCGTCGGCGGC	GGGCTCACCCCCAAACCTGCAG	Polymorphic	1	300	60	42.86
RM 250	2	GGTTCAAACCAAGCTGATCA	GATGAAGGCCTTCCACGCAG	Polymorphic	3	200–500	51	29.52
RM 256	8	GACAGGGAGTGATTGAAGGC	GTTGATTTCGCCAAGGGC	Polymorphic	1	65	59	82.86
RM 285	9	CTGTGGGCCCAATATGTCAC	GGCGGTGACATGGAGAAAG	Polymorphic	2	150–200	55	52.86
RM 288	9	CCGGTCAGTTCAAGCTCTG	ACGTACGGACGTGACGAC	Polymorphic	1	170	62	51.43
RM 291	5	GTTGCACTACGTATTCTGAG	GATCCAGATAAATGAGGCAC	Polymorphic	1	200	58	60.00
RM 294	1	TTGGCCTAGTGCCTCCAATC	GAGGGTACAACTTAGGACGCA	Polymorphic	2	180–200	62	75.71
RM 311	10	TGGTAGTATAGGTACTAAACAT	TCCTATACACATACAAACATAC	Polymorphic	1	300	62	34.29
RM 314	6	CTAGCAGGAACTCCTTTCAGG	AACATTCCACACACACACGC	Polymorphic	1	170	62	31.43
RM 321	9	CCAACACTGCCACTCTGTTC	GAGGATGGACACCTTGATCG	Polymorphic	1	200	62	48.57
RM 327	2	CTACTCCTCTGTCCCTCCTCTC	CCAGCTAGACACAATCGAGC	Polymorphic	1	200	64	45.71
RM 332	11	GCGAAGGCGAAGGTGAAG	CATGAGTGATCTCACTCACCC	Polymorphic	1	180	62	40.00
RM 342	8	CCATCCTCCTACTTCAATGAAG	ACTATGCAGTGGTGTCACCC	Polymorphic	1	180	62	31.43
RM 434	9	GCCTCATCCCTCTAACCCTC	CAAGAAAGATCAGTGCGTGG	Polymorphic	1	185	62	97.14
RM 460	9	TGATCGACAGCGTTCTTGAC	GCCTGGCCCACATAATTAAG	Polymorphic	1	300	62	14.29
RM 469	6	AGCTGAACAAGCCCTGAAAG	GACTTGGGCAGTGTGACATG	Monomorphic	1	85	62	100.00
RM 3134	3	GCAGGCACAAAAGCAAAGAG	AGGTGAAGGTGCATTGTGTG	Polymorphic	1	185	62	28.57
RM 3872	3	GGAAGAAAGGATCTATATCA	TACGATTTGTTTAAGTTCAA	Polymorphic	1	150	62	31.43
RM 6250	4	AACCTACGTTACCCTGCACG	GGCTCATGAGTTTCAGAGGC	Polymorphic	1	180	52	22.86
RM 7376	12	TCACCGTCACCTCTTAAGTC	GGTGGTTGTGTTCTGTTTGG	Polymorphic	1	200	62	40.00
RM 10022	1	CCTCCATAGAGTAAGGTTTGCATGG	CCTCCTCCTCTGTCTTTCTCTGC	Polymorphic	2	200–400	56	68.57
RM 16655	4	CCTTGGAAGCTGGAACTTCACC	GGCTCTTAGGTTAGATCCCACACG	Polymorphic	1	200	60	88.57
RM 23835	9	TTCCGCTGTTTCTCTTCTTGTGC	CTGGTTCTGCTGGTTCTGTAGTTGG	Polymorphic	1	200	58	54.29
ISSR1	–	(GGC)5AT		Polymorphic	2	400–2000	66	41.43
ISSR2	–	(AAG)5GC		Polymorphic	3	150–2000	48	31.43
ISSR3	–	(AAG)5TG		Polymorphic	3	150–2500	50	42.86
ISSR4	–	(AAG)5CC		Polymorphic	3	150–2500	50	43.81
ISSR5	–	(AGC)5CA		Polymorphic	2	150–1000	62	54.29
ISSR6	–	(AGC)5CG		Polymorphic	3	500–3500	52	43.81
ISSR7	–	(GGC)5TA		Polymorphic	5	150–3000	66	51.43
ISSR8	–	(AGC)5GA		Polymorphic	4	250–1500	62	40.71
ISSR9	–	(AAG)5CG		Polymorphic	3	400–2900	52	36.19
ISSR10	–	CCA(GTG)4		Polymorphic	2	300–2000	60	78.57

The primers for the SSR and ISSR markers were designed following article reference nos.^27,28^.

**Table 4:** Polymorphism information of the thirty-five genotypes generated using microsatellite markers.

Markers	H	PIC	E	H. Av	MI	D	R
RM1	0.345	0.285	0.222	0.009	0.002	0.955	0.444
RM23	0.277	0.239	0.500	0.002	0.001	0.973	1.00
RM38	0.345	0.285	0.222	0.009	0.002	0.955	0.444
RM108	0.054	0.052	0.972	0.001	0.001	0.055	0.055
RM114	0.313	0.264	1.611	0.004	0.007	0.353	0.777
RM159	0.208	0.186	3.527	0.001	0.005	0.222	0.944
RM165	0.461	0.354	0.722	0.006	0.004	0.872	1.22
RM169	0.461	0.354	1.277	0.006	0.008	0.595	0.555
RM172	0.475	0.362	0.388	0.013	0.005	0.855	0.777
RM195	0.493	0.371	0.444	0.013	0.006	0.809	0.888
RM250	0.431	0.338	0.944	0.003	0.003	0.902	0.777
RM256	0.313	0.264	0.805	0.008	0.007	0.355	0.388
RM285	0.496	0.373	1.083	0.006	0.007	0.710	1.611
RM288	0.500	0.375	0.500	0.013	0.006	0.757	1.00
RM291	0.486	0.367	0.583	0.013	0.007	0.666	0.833
RM294	0.360	0.295	1.527	0.005	0.007	0.419	0.944
RM311	0.444	0.345	0.333	0.012	0.004	0.895	0.666
RM314	0.424	0.334	0.305	0.011	0.003	0.912	0.611
RM321	0.498	0.374	0.472	0.013	0.006	0.784	0.944
RM327	0.498	0.374	0.472	0.013	0.006	0.784	0.944
RM332	0.475	0.362	0.388	0.013	0.005	0.855	0.777
RM342	0.424	0.334	0.305	0.011	0.003	0.912	0.611
RM434	0.054	0.052	0.972	0.001	0.001	0.055	0.055
RM460	0.277	0.239	0.166	0.007	0.001	0.976	0.333
RM469	0	0	1	0	0	0	0
RM3134	0.424	0.334	0.305	0.011	0.003	0.912	0.611
RM3872	0.424	0.334	0.305	0.011	0.003	0.912	0.611
RM6250	0.345	0.285	0.222	0.009	0.002	0.955	0.444
RM7376	0.475	0.362	0.388	0.013	0.005	0.855	0.777
RM10022	0.424	0.334	1.388	0.005	0.008	0.520	0.777
RM16655	0.197	0.178	0.888	0.005	0.004	0.212	0.222
RM23835	0.493	0.371	0.555	0.013	0.007	0.698	0.888
ISSR1	0.481	0.365	0.805	0.006	0.005	0.841	1.611
ISSR2	0.431	0.338	0.944	0.003	0.003	0.902	1.888
ISSR3	0.489	0.369	1.277	0.004	0.005	0.820	2.111
ISSR4	0.489	0.369	1.277	0.004	0.005	0.820	1.888
ISSR5	0.498	0.374	1.055	0.006	0.007	0.724	1.888
ISSR6	0.491	0.370	1.305	0.004	0.005	0.812	1.055
ISSR7	0.499	0.374	2.555	0.002	0.007	0.740	3.222
ISSR8	0.486	0.367	1.666	0.003	0.005	0.888	3.333
ISSR9	0.456	0.352	1.055	0.004	0.004	0.878	2.000
ISSR10	0.360	0.295	1.527	0.005	0.007	0.419	0.944

*H: *heterozygosity index, *PIC: *polymorphic information content, *E: *effective multiplex ratio, *H:. av *arithmetic mean of H, *MI: *marker index, *D: *discriminating power, *R: *resolving power.

The evaluation of genetic diversity for characterization of these heirloom cultivars is necessary and relatively easy due to the availability of nearly saturated molecular map in case of rice^27,28,35^. SSRs are interspersed throughout the genome and known as mono-locular, co-dominant, highly informative and easy to analyze marker system where as ISSR markers relies on inter tandem repeats of di, tri, tetra or penta nucleotides found at microsatellite loci and gives an array of amplified products. Purine rich ISSR markers have been proved to be very useful in determining genetic relationship between diverse population^36–38^. In the present study we have performed genetic diversity analysis of locally grown scented landraces using ISSR and SSR markers and based on the results the cultivars were clustered. The results of the marker analysis emphasize that each of these cultivars are distinct from the other and the population under study is genetically diverse. It is noteworthy that the marker-based clustering grouped the genotypes according to the yield potential which proves the accuracy and usefulness of microsatellite marker system in determining purity and phylogeny of the germplasm. This natural population of aromatic cultivars with such wide genetic variability may prove to be good resources for excellent quality traits and disease tolerance. These cultivars were further analyzed for the grain quality attributes and tolerance to destructive blast disease.

### Analysis of nutritional variability in selected rice genotypes

Nutritional parameters like Total Soluble Sugar (TSS), Reducing Sugar (RES), Non- Reducing Sugar (NRS), Starch (STA), Amylose (AMY), Resistant Starch (RS), Glycemic Index (GI), Protein (PRO), Antioxidant properties (ANT) and Aroma (ARO) has been measured for thirty-five selected genotypes (Table 5). The TSS content of the genotypes varied from 88.89% (‘Chakhao Sampark’) to 37.44% (‘Tulsibhog’) whereas average starch content of these genotypes ranged from 85.45% (‘Sadanunia’) to 45.86% (‘Konkanijoha’). The wide variability in carbohydrate content prompted us to measure the amylose content of these genotypes as the ratio of amylose and amylopectin content determines the stickiness and flakiness of the rice after cooking. Higher amylose content was found in KNS-2-1-1 (31.27%), ‘Tulsibhog’ (29.10%) and ‘Kabra’ (28.77%) etc. whereas low amylose was found in genotypes like ‘Chakhao’ selections, ‘Kalojeera’, ‘Konkanijoha’, ‘Gobindabhog’, ‘Kalturey’ etc. Good resistant starch (RS) content in ‘Kataribhog’ (2.25%), ‘Chakhao Sampark’ (2.22%), and KNS-2-1-1 (2.11%) were found. Although most of the genotypes showed an average GI value of more than 60%, few cultivars like ‘Dubarikomal (54.77%), ‘A-1-1’ (58.67%), ‘KNS-2B-S1’ (55.08%), ‘TPUR-B-1-IET 28104’ (55.63%) exhibited quite encouraging low glycemic index. Powdered ‘Kataribhog’ grains were found to have remarkable GI, as low as 45.72% by initial analysis which is even lower than the GI of recommended rice varieties for diabetic patients^39^. The protein content of the genotypes was varying from 4.11% in ‘Rampha’ and was highest in 9.47% in ‘Dharmaphou’. When the total antioxidant activity was evaluated for powdered grains of these genotypes all the genotypes with black pericarp has exhibited the highest antioxidant potential (‘Chakhao sel-2’, ‘Chakhao sel-3’, ‘Chakhao Sampark’, ‘Chakhao Poiterin’ and ‘Kalshipa’). Apart from black genotypes four indigenous cultivars ‘Sadanunia’, ‘Kabra’, ‘Kalturey’, ‘Rangakomal’ have exhibited very high antioxidant activity comparable to the black varieties. All the varieties exhibited good to very good aroma.

**Table 5:** Variation in different nutritional attributes of selected indigenous aromatic rice genotypes.

Genotype	Total Soluble sugar (g/100 g)	Reducing sugar (g/100 g)	Non-reducing sugar (g/100 g)	Starch (%)	Amylose (%)	Amylopectin (%)	Resistant starch (%)	Glycemic index (%)	Protein (%)	Antioxidant activity (IC_50_) (µg/ml)	Aroma
Tulaipanji	53.96 ± 1.71^g–n^	2.47 ± 0.29^a–d^	0.77 ± 0.15^g–k^	68.99 ± 1.46^i–o^	20.43 ± 1.88^g–k^	48.56 ± 0.42^f–g^	0.87 ± 0.09^h–m^	67.38 ± 0.09^b-d^	8.30 ± 0.10^c–e^	873.67 ± 16.04^o–q^	3
Radhatilak	80.58 ± 3.07^a–d^	2.58 ± 0.41^a–c^	0.85 ± 0.12^f–k^	66.27 ± 1.73^k–p^	23.43 ± 0.94^c–i^	42.83 ± 0.79^g–i^	0.90 ± 0.01^h–l^	68.21 ± 0.05^b,c^	8.91 ± 0.10^a-c^	1231.75 ± 15.87^m–o^	2
Kalshipa	42.03 ± 2.16^m,n^	2.46 ± 0.55^a–d^	0.95 ± 0.14^e–j^	83.21 ± 0.09^a,b^	26.93 ± 1.17^a–e^	56.27 ± 1.27^d–f^	0.92 ± 0.20^g–k^	66.70 ± 0.02^b-e^	6.10 ± 0.03^h–k^	314.72 ± 65.23^s^	1
Rongakomal	88.24 ± 1.37^a^	2.40 ± 0.21^b–d^	1.35 ± 0.16^b–e^	83.17 ± 0.69^a,b^	23.10 ± 0.94^d–i^	60.07 ± 0.24^a–d^	0.73 ± 0.05^h–m^	64.67 ± 0.03^c–h^	5.39 ± 0.10^k–n^	408.76 ± 22.64^s^	2
Sadanunia	45.02 ± 0.91^j–n^	1.96 ± 0.01^c–e^	1.04 ± 0.23^c–h^	85.45 ± 1.22^a^	19.43 ± 1.88^h–l^	66.01 ± 0.65^a,b^	0.77 ± 0.09^h–m^	60.49 ± 0.06^i–j^	5.82 ± 0.14^i–l^	338.79 ± 34.85^s^	2
Tulsibhog	37.44 ± 3.42^n^	1.57 ± 0.20^e–g^	0.76 ± 0.24^g–k^	82.29 ± 0.13^a–c^	29.10 ± 2.82^a,b^	53.18 ± 2.68^e–f^	0.97 ± 0.05^f–j^	65.02 ± 2.09^b–h^	8.65 ± 0.18^b–d^	2028.44 ± 42.14^g–j^	2
Kalturey	43.16 ± 2.62^k–n^	1.10 ± 0.34^f–j^	0.69 ± 0.02^g–k^	63.60 ± 1.76^m–p^	9.93 ± 0.23^n,o^	53.66 ± 1.99^c–f^	0.93 ± 0.03^g–k^	63.70 ± 0.22^b–i^	3.86 ± 0.25^q^	594.64 ± 10.88^q–s^	3
Kataribhog	64.77 ± 1.48^c–j^	1.59 ± 0.32^e–j^	0.77 ± 0.17^g–k^	54.57 ± 3.59^q,r^	20.43 ± 5.65^g–k^	34.13 ± 9.25^i–k^	2.25 ± 0.01^a^	45.72 ± 0.79^m^	6.43 ± 0.07^h,i^	1583.68 ± 53.52^k–m^	2
A-1-1	77.43 ± 2.50^a–e^	1.72 ± 0.72^d–f^	0.66 ± 0.13^g–k^	66.88 ± 1.29^j–p^	13.26 ± 2.59^m,n^	53.61 ± 3.89^c–f^	1.79 ± 0.01^a–d^	58.67 ± 0.27^j,k^	9.34 ± 0.14^a,b^	1065.78 ± 54.27^o,p^	1
Dubarikomal	63.65 ± 2.39^c–l^	0.94 ± 0.71^f–j^	0.89 ± 0.09^f–k^	74.76 ± 1.78^c–j^	7.60 ± 0.70^o^	67.16 ± 2.49^a^	1.91 ± 0.02^a–c^	54.77 ± 0.07^l^	9.19 ± 0.07^a,b^	1535.27 ± 73.37^k–m^	2
Darmaphou	62.52 ± 1.48^d–n^	3.21 ± 0.28^a^	0.99 ± 0.20^d–y^	70.22 ± 1.48^h–o^	27.93 ± 1.64^a–d^	42.28 ± 0.16^g–i^	1.82 ± 0.01^a–d^	62.70 ± 0.17^f–i^	9.47 ± 0.03^a^	2216.74 ± 91.96^f–h^	1
Dudheswar	69.45 ± 2.62^a–i^	3.10 ± 1.05^a,b^	0.79 ± 0.22 g^-k^	79.44 ± 2.45^a–f^	13.27 ± 1.17^m,n^	66.16 ± 1.27^a,b^	1.44 ± 0.01^d–f^	63.66 ± 0.08^b–i^	5.87 ± 0.14^i,l^	3241.72 ± 39.58^a^	1
Dudhkalam	68.41 ± 2.96^a–j^	1.50 ± 0.55^e–g^	0.49 ± 0.12^j,k^	49.94 ± 5.91^r,s^	21.93 ± 1.17^e–i^	28.01 ± 4.73^k^	1.39 ± 0.03^d–g^	63.40 ± 0.08^b–i^	7.68 ± 0.10^e,f^	2489.88 ± 21.02^d–f^	1
Ranpha	55.42 ± 0.56^f–n^	1.16 ± 0.05^e–i^	0.51 ± 0.11^i–k^	82.93 ± 7.07^a,b^	25.10 ± 1.88^b–g^	57.82 ± 5.18^a–f^	1.16 ± 0.03^e–h^	65.33 ± 0.03^b–h^	4.11 ± 0.10^q,p^	2979.73 ± 29.25^a–c^	3
Gobindabhog	53.65 ± 4.67^j–n^	0.87 ± 0.30^f–j^	0.60 ± 0.11^h–k^	73.39 ± 5.68^d–m^	7.43 ± 4.71^o^	65.95 ± 0.96^a,b^	1.05 ± 0.06^e–i^	66.14 ± 0.11^b–f^	6.77 ± 0.10^g,h^	1152.79 ± 45.11^n–p^	3
Konkanijoha	83.97 ± 0.79^a–c^	0.91 ± 0.23^f–j^	0.44 ± 0.20^k^	45.86 ± 4.40^s^	14.77 ± 1.41^l–m^	31.09 ± 5.81^j–k^	0.97 ± 0.04^f–j^	65.08 ± 0.13^b–h^	9.29 ± 0.22^a,b^	1859.23 ± 20.60^i–k^	3
Kalokhasa	67.03 ± 4.67^b–i^	0.49 ± 0.08^i,j^	0.41 ± 0.02^k^	60.06 ± 2.27^p,q^	24.93 ± 2.59^b–h^	35.12 ± 4.86^i–k^	1.40 ± 0.04^d–g^	62.25 ± 0.01^g–j^	8.63 ± 0.14^b-d^	1740.15 ± 39.52^j–l^	1
Kabra	64.21 ± 5.24^c–k^	0.80 ± 0.14^g–j^	0.60 ± 0.14^h–k^	76.53 ± 2.38^b–i^	28.77 ± 0.47^a–c^	47.76 ± 2.85^f–h^	1.14 ± 0.02^e–h^	66.45 ± 0.06^b–f^	4.75 ± 0.07^l,p^	510.16 ± 67.34^r,s^	3
Radhunipagol	57.52 ± 11.51^e–n^	0.73 ± 0.17^g–j^	0.64 ± 0.01^g–k^	78.49 ± 4.96^a–g^	23.10 ± 1.41^g–i^	55.38 ± 6.37^c–f^	1.46 ± 0.02^c–e^	58.86 ± 0.07^j–k^	7.71 ± 0.07^e,f^	1875.15 ± 8.13^h–k^	3
Kalojeera	61.87 ± 1.93^d–n^	0.99 ± 0.16^f–j^	0.75 ± 0.12^g–k^	80.96 ± 0.30^a–d^	19.10 ± 0.47^i–l^	61.86 ± 0.17^a–d^	0.60 ± 0.07^i–m^	74.36 ± 0.13^a^	5.74 ± 0.25^i,l^	1777.82 ± 40.00^j–l^	3
Kalonunia	50.74 ± 3.99^h–m^	1.02 ± 0.28^f–j^	0.59 ± 0.02^h–k^	72.49 ± 1.94^e–m^	22.77 ± 3.29^d–i^	49.71 ± 5.24^e–g^	0.75 ± 0.01^h–m^	66.85 ± 0.13^b–e^	7.15 ± 0.14^f,g^	2147.88 ± 14.89^f–i^	3
Chakhao sel-1	86.47 ± 1.59^a,b^	0.87 ± 0.57^f–j^	1.42 ± 0.36^b–d^	76.55 ± 2.08^d–i^	19.10 ± 4.71^i–l^	57.45 ± 6.80^a–f^	1.65 ± 0.01^b–d^	61.55 ± 0.19^h–j^	5.41 ± 0.21^j–m^	2679.21 ± 21.17^c,d^	1
Chakhao Poiterein	59.37 ± 6.61^e–m^	0.77 ± 0.05^g–j^	1.47 ± 0.57^b,c^	63.39 ± 11.80^o,p^	7.77 ± 2.82^o^	55.62 ± 14.62^c–f^	0.40 ± 0.04^m^	60.81 ± 0.12^i,j^	4.88 ± 0.10^m–o^	596.99 ± 68.78^q–s^	3
Chapka Chakhao	75.74 ± 1.02^a–f^	0.44 ± 0.26^i,j^	1.51 ± 0.09^b,c^	79.34 ± 1.80^a–g^	19.93 ± 0.70^g–l^	59.40 ± 2.51^a–e^	0.82 ± 0.01^h–n^	64.94 ± 0.12^b–h^	4.60 ± 0.07^m–p^	823.74 ± 103.55^p–r^	1
Chakhao-2	74.53 ± 2.73^a–h^	0.41 ± 0.02^i,j^	1.32 ± 0.12^c–f^	65.94 ± 0.20^l–b^	11.27 ± 0.23^m–o^	54.67 ± 0.44^c–f^	0.47 ± 0.02^k–m^	64.90 ± 0.23^b–h^	4.90 ± 0.21^l–o^	386.43 ± 76.83^s^	1
Chakhao-3	58.00 ± 1.71^e–n^	0.28 ± 0.08^j^	1.12 ± 0.25^c–g^	71.27 ± 1.11^g–n^	7.93 ± 0.70^o^	63.33 ± 1.81^a–c^	0.47 ± 0.08^k–m^	63.74 ± 0.13^b–i^	5.23 ± 0.10^k–n^	558.58 ± 33.75^q,r,s^	1
Chakhao Sampark	88.89 ± 1.14^e–n^	0.31 ± 0.04^i,j^	1.39 ± 0.24^b–e^	82.78 ± 2.87^a,b^	24.93 ± 4.00^b–y^	62.00 ± 1.71^a–f^	2.22 ± 0.03^d–g^	59.55 ± 0.19^b–f^	9.21 ± 0.11^d,e^	411.81 ± 3.38^o,p^	1
KNS-2′-1	56.55 ± 1.02^e–n^	1.40 ± 0.09^e–h^	0.69 ± 0.08^g–k^	78.50 ± 1.08^a–g^	21.10 ± 1.41^f–j^	57.40 ± 2.50^a–f^	0.65 ± 0.01^i–m^	74.73 ± 0.22^a^	7.38 ± 0.10^f,g^	3131.05 ± 91.85^a,b^	2
KNS-3′-1 Uttar Sugandhi (IET 24,616)	54.37 ± 5.01^f–n^	0.95 ± 0.22^f–j^	0.86 ± 0.22^f–k^	74.12 ± 4.40^g–k^	19.93 ± 1.17^g–l^	54.19 ± 5.58^c–f^	0.55 ± 0.01^i–m^	72.80 ± 0.05^a^	6.13 ± 0.28^h–j^	2829.75 ± 66.25^b–d^	2
KNS-2-1-1	58.89 ± 4.33^e–m^	0.77 ± 0.09^g–j^	0.69 ± 0.12^g–k^	80.52 ± 4.17^a–e^	31.27 ± 3.06^a^	49.25 ± 1.10^e–g^	2.11 ± 0.01^a,b^	67.59 ± 0.02^b,c^	5.44 ± 0.10^j–m^	2281.55 ± 12.51^e–g^	1
KNS-2B-S1	85.66 ± 1.59^a,b^	0.59 ± 0.13^h–j^	1.80 ± 0.07^a,b^	77.40 ± 0.27^d–h^	22.27 ± 0.70^e–i^	55.13 ± 0.42^c–f^	1.99 ± 0.01^a,b^	55.08 ± 0.14^l^	4.80 ± 0.07^l–p^	1455.50 ± 12.44^l–n^	2
T4M-3-5	47.92 ± 0.67^i–n^	0.35 ± 0.07^i,j^	2.02 ± 0.09^a^	77.14 ± 1.43^d–h^	16.27 ± 0.23^j–n^	60.87 ± 1.20^a–d^	0.64 ± 0.01^i–m^	66.03 ± 0.16^b–g^	8.93 ± 0.21^a–c^	1572.50 ± 103.23^k–m^	2
TSP6-M3-4	59.29 ± 5.35^e–m^	0.61 ± 0.02^h–j^	1.81 ± 0.24^a,b^	72.03 ± 1.34^f–m^	15.27 ± 0.23^k–n^	56.75 ± 1.58^e–f^	0.42 ± 0.03^l,m^	68.54 ± 0.20^b^	5.23 ± 0.18^l–m^	1145.20 ± 8.06^n–p^	1
TSPM-3-1 TPUR-B-1(IET 28,104)	61.79 ± 0.67^d–n^	0.60 ± 0.02^h–j^	0.87 ± 0.48^f–k^	73.68 ± 0.71^d–l^	21.10 ± 1.41^f–j^	52.58 ± 2.13^d–f^	0.49 ± 0.11^k–m^	55.63 ± 0.03^k,l^	5.77 ± 0.14^i–l^	1605.10 ± 16.68^k,l^	2
T6M-3-3	42.68 ± 3.07^l–n^	0.45 ± 0.13^i,j^	0.70 ± 0.31^g–k^	65.47 ± 0.78^m–p^	26.60 ± 1.64^a–f^	38.86 ± 2.43^h–j^	0.57 ± 0.07^i–m^	66.21 ± 0.15^b–f^	4.49 ± 0.07^o,p,q^	2580.75 ± 123.10^d,e^	2
CV^##^	9.97	20.32	14.96	3.25	8.34	5.60	13.25	1.74	3.31	7.10	
F value	**	**	**	**	**	**	**	**	**	**	

Different letters in the same line means statistical difference (p < 0.05) by Duncan test. CV means coefficient of variance.

Statistical significance was measured by F test, **means values are statistically significant at p < 0.01.

**Figure 4:**
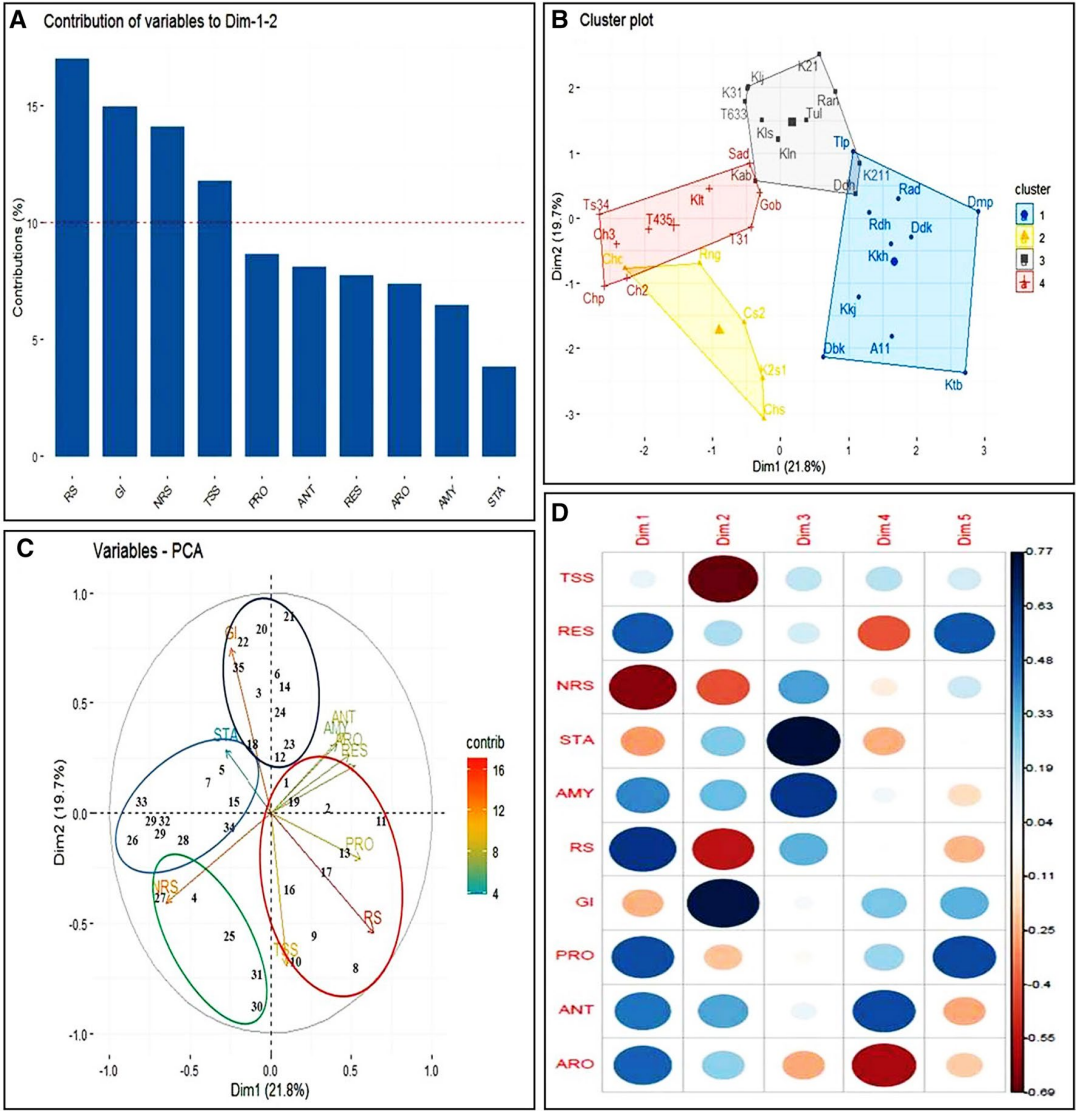
PCoA biplot of thirty-five genotypes based on their important nutritional attributes. All the analysis was performed using R software^31^, version 3.5.1, Patched (2018–07-02 r74950) Platform: x86_64-w64mingw32/x64 (64-bit) (https://www.R-project.org/). (**A**) Contribution of various nutritional traits based on the mean values for the grouping of the rice genotypes. The Y-axis depicts the contribution of the parameters in percentage (%). (**B**) Cluster analysis of the rice genotypes, the X and Y axis represent the PC1 (dim1) and PC2 (dim2) and the percentage (%) of total variation defined by PC1 and PC2. (**C**) PCoA biplot exhibiting the grouping of the genotypes as well as the nutritional characters. The X and Y axis represent the PC1 (dim1) and PC2 (dim2) and the percentage (%) of total variation defined by PC1 and PC2. (**D**) Dimension wise distribution of the nutritional characters contributing towards the clustering of the genotypes. The genotypes are denoted numerically or in short as follows 1. ‘Tulaipanji’ (Tlp), 2. ‘Radhatilak’ (Rad), 3. ‘Kalshipa’ (Kls), 4. ‘Rangakomal’ (Rng), 5. ‘Sadanunia’ (Sad), 6. ‘Tulsibhog’ (Tul), 7. ‘Kalturey’ (Klt), 8. ‘Kataribhog’ (Ktb), 9. ‘A-1-1’ (A11), 10. ‘Dubarikomal’ (Dbk), 11. ‘Dharmaphou’ (Dmp), 12. ‘Dudheswar’ (Ddh), 13. ‘Dudhkalam’ (Ddk), 14. ‘Rampha’ (Ran), 15. ‘Gobindabhog’ (Gob), 16. ‘Konkanijhoha’ (Kkj), 17. ‘Kalokhasa’ (Kkh), 18. ‘Kabra’ (Kab), 19. ‘Radhunipagol’ (Rdh), 20. ‘Kalojeera’ (Klj), 21. ‘KNS-2′-1’ (K2′1), 22. ‘KNS-3′-1’ (K3′1), 23. ‘KNS-2-1-1’ (K211), 24. ‘Kalonunia’ (Kln), 25. ‘Chakhao sel-1’ (Cs1), 26. ‘Chakhao Poiterin’ (Chp), 27. ‘Chapka Chakhao’ (Chc), 28. ‘Chakhao sel-2’ (Ch2), 29. ‘Chakhao sel-3’ (Ch3), 30. ‘Chakhao Sampark’ (Chs), 31. ‘KNS-2B-S1’ (K2s1), 32. ‘T4M-3-5’ (T435), 33. ‘TSP6-M3-4’ (TS34), 34. ‘TPUR-B-1’ (TSPM-3-1) (T31), 35. ‘T6M-3-3’ (T633). The attributes are described in short form as follows. *TSS *total soluble sugar, *RES *reducing sugar, *NRS *non-reducing sugar, *STA *starch, *AMY *amylose, *RS *resistant starch, *GI *Glycemic Index, *PRO *protein, *ANT *antioxidant, *ARO *aroma.

Based on the nutritional parameters, the genotypes were clustered statistically. Resistant Starch (RS), Glycemic Index (GI), Non-Reducing Sugar (NRS) and Total Soluble Solids (TSS) were found to be contributing to the variability of the cultivars (Fig. 4A, Supplementary information 3a). Both the cluster plot and Principal Component Analysis divided the genotypes in four distinct clusters (Fig. 4B). The first cluster with genotypes like ‘Kataribhog’, ‘Radhatilak’, ‘Radhunipagol’, ‘Dudhkalam’, ‘Kalokhasa’, ‘Dubarikomal’, ‘Dharmaphou’, ‘Konkanijoha’ etc. were found to be low in Glycemic index (GI) and high in Resistant Starch (RS). The second cluster were constituted by ‘Rampha’, ‘Tulaipanji’, ‘Kalonunia’, ‘Dudheswar’, ‘Kalshipa’, ‘Kalojeera’, ‘KNS-2′-1’, ‘KNS-3′-1’, ‘T6M-3-3’ have relatively high GI values. The third group constituted of the photo insensitive lines of ‘Tulaipanji’ like ‘TSP6-M3-4’, ‘T4M-3-5’, ‘TPUR-B-1(IET 28104)’ along the black varieties like ‘Chakhao sel-3’, ‘Chakhao sel-2’, ‘Chakhao Poiterin’ and two popular genotypes ‘Sadanunia’ and ‘Gobindabhog’. All these genotypes exhibited high starch content. PCA of the variables were performed and Resistant Starch (RS), Total Soluble Sugar (TSS) and Glycemic Index (GI) were found to be significantly contributing in the grouping of the genotypes. The Strach content (STA), Glycemic Index (GI) were found in opposite dimension to Resistant Starch (RS) in PCoA biplot (Fig. 4C). Correlation analysis suggested that the RS and the GI are negatively correlated whereas Protein (PRO), Antioxidant activity (ANT) and Aroma (ARO) were found positively correlated in these genotypes (Fig. 4D).

Considering the high nutritional and ethno-medicinal values of local land races, the present study has special merits. Starch is the major contributor which determines the appearance of rice after cooking and its consistency^40^. The starch granules are aggregation of linear amylose chain and highly branched amylopectin fractions^41^. The proportion of amylose and amylopectin has profound effect on the physicochemical properties of rice like stickiness, water absorption, volume expansion, hardness and texture of rice^40^. The amylose content of Pusa Basmati was found to be less when compared with ‘Gobindabhog’^42^. Along with good cooking quality, modern health-conscious consumers prefer rice with high medicinal value. Due to the lifestyle diseases like obesity, Type II diabetes mellitus, hypertension, etc. the popularity of rice is declining in many countries as white starch is considered to be one of the detrimental source of energy. Very few studies have been conducted for the estimation of health benefitting GI and RS of the Indian scented rice. Rice generally contains less than 3% of type 5 resistant starch^43^. Resistant starch produces zero calories on digestion, and offers health benefit for diabetic patients. Many of the previous work has also reported strong negative correlation of RS and GI in rice cultivars even after cooking^44^ where as high amylose content rice cultivars have been reported to have low GI^24,45^. GI of Pusa Basmati 1121 has been reported as 58.41% which has been shown to reduce by steaming of the paddy or by different cooking treatments^46,47^. As compared to basmati; the land races like, ‘Kataribhog’, ‘A-1-1’, ‘Dubarikomal’, ‘KNS-2B-S1’, ‘TPUR-B-1 (IET 28104)’ etc. demonstrates very good health parameters with low GI and high RS. Although black rice has been known to have high antioxidant activity but the local genotypes like ‘Sadanunia’, ‘Rangakomal’, ‘Kalshipa’ etc. has shown promising antioxidant activity which is comparable with black cultivars. Non-basmati aromatic cultivars from Bangladesh have been evaluated for their phenol and flavonoid content and have been proved to have good antioxidant potential^48^. Cultivars like ‘Gopalbhog’, ‘Gobindabhog’, ‘Badshabhog’ etc. has been found to have good nutritional value^49^. Altogether the results indicate that the local cultivars of these region have very good quality traits and can be popularized as healthy rice or may be utilized in breeding programs for quality enhancement.

### Screening of the selected aromatic genotypes against rice blast disease

**Figure 5:**
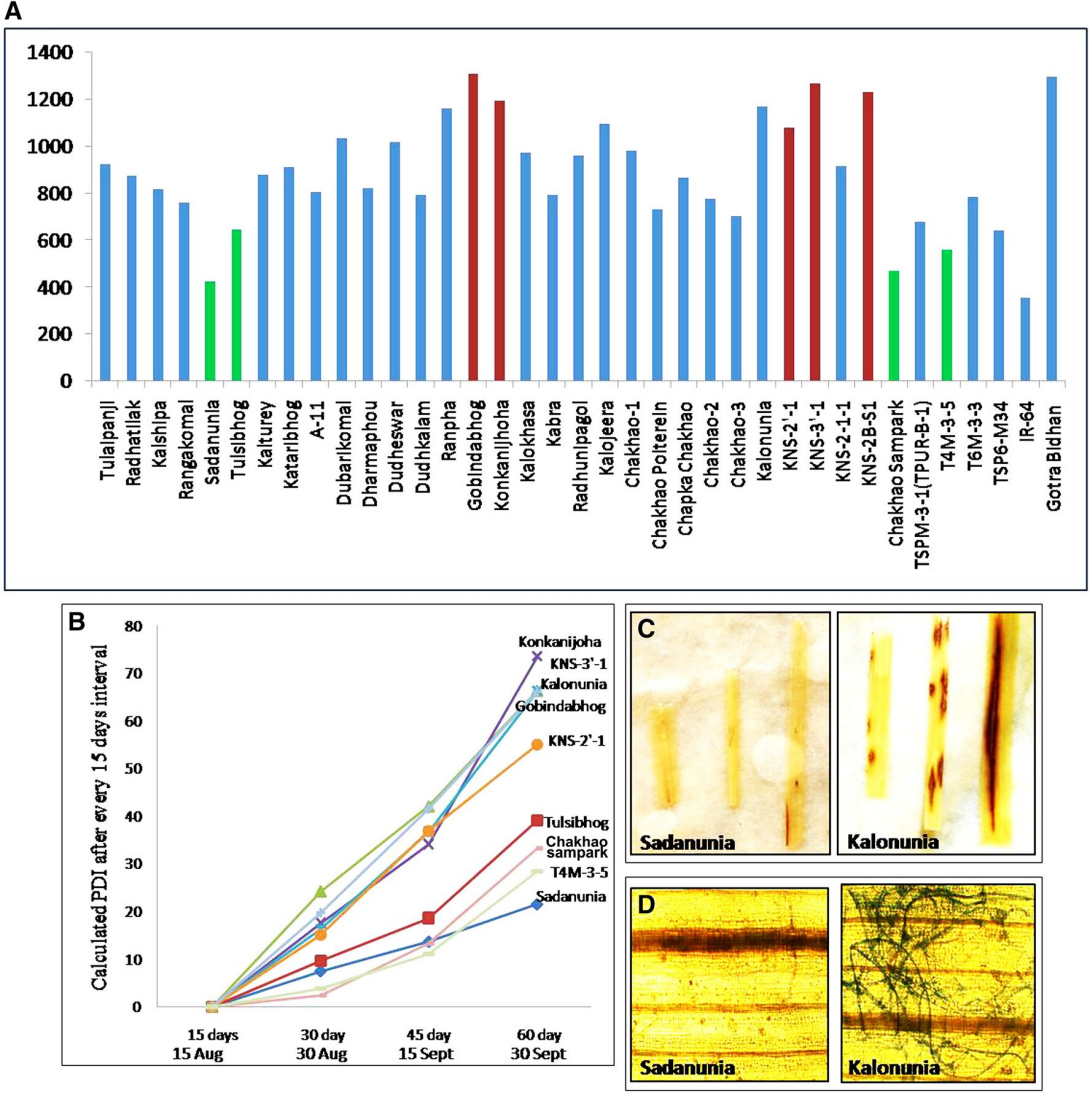
Screening of the local landraces for the occurrence of the leaf last disease. (**A**) Calculated AUDPC of all the genotypes from 2 year’s field trial and scoring of the plants for disease symptoms. (**B**) PDI of some highly susceptible and tolerant cultivars between mid of August to early October. (**C**) Time lapse photography of the leaves from susceptible (‘Kalonunia’) and tolerant (‘Sadanunia’) cultivars after inoculation from purified blast fungus. (**D**) Microscopic image of hyphal growth in susceptible (‘Kalonunia’) and tolerant (‘Sadanunia’) cultivars.

The sub-Himalayan Terai region is hot spot for blast disease and the meteorological data indicates that mid-September to late October is the most suitable time for the outbreak of the disease (Supplementary dataset 1). The AUDPC (Area Under Disease Progress Curve) indicates that cultivars like ‘Sadanunia’, ‘Tulsibhog’, ‘Chakhao Sampark’, ‘T4M-3-5’ etc. exhibited extreme tolerance for leaf blast disease whereas ‘Gobindabhog’, ‘Konkanijoha’, ‘Kalonunia’, ‘Rampha’, ‘KNS-2B-S1’, ‘KNS-3′-1’ etc. were found to be highly susceptible to leaf blast under natural conditions (Table 6, Fig. 5A). From the PDI values it has been noticed that the disease progresses very rapidly in the month of September for all the genotypes (Fig. 5B). The distinctness of the tolerant versus susceptible cultivars can be observed during this period. *In-vitro* disease progression assay suggested that the susceptible cultivars develop symptoms after 24 h of inoculation whereas the tolerant cultivars did not develop symptoms even after 48 h of inoculation (Fig. 5C). Time lapse microscopy of susceptible cultivars showed spore germination and hyphae development within 24 h post inoculation in susceptible cultivars like ‘Gobindabhog’ and ‘Konkanijoha’ unlike tolerant cultivars ‘Sadanunia’, ‘T4M-3-5’ etc. (Fig. 5D). Both in field experimets and in-vitro experiments represent discreet difference in disease severity among the selected genotypes.

**Table 6:** Variation in plant morphological and disease related characters of selected indigenous aromatic rice genotypes.

Genotypes	Plant height (cm)	Tiller number	Lower leaf angle (°)	Middle leaf angle(°)	Upper leaf angle(°)	Lesion no. in lower leaf	Lesion no. in middle leaf	Lesion no. in upper leaf	Lesion type	Lesion size (mm^2^)	Sporulation centre	AUDPC for blast
Tulaipanji	110.37 ± 0.52^c–g^	38.10 ± 4.52^a^	29.23 ± 3.29^b–f^	15.33 ± 5.13^f^	9.55 ± 2.05^e–f^	4.07 ± 2.12^a,b^	7.43 ± 0.80^a–c^	11.68 ± 2.19^a^	1.31 ± 0.01^n^	1.26 ± 0.006^o,p^	0.34 ± 0.015^i–k^	922.01 ± 130.77^d–k^
Radhatilak	109.22 ± 2.61^d–g^	33.62 ± 2.94^a,b^	42.07 ± 8.67^a^	20.42 ± 3.41^b–f^	9.50 ± 0^f^	3.37 ± 4.43^a,b^	4.47 ± 4.90^a–e^	5.50 ± 4.24^b–d^	1.28 ± 0.01^o,p^	1.41 ± 0.010^i,j^	0.34 ± 0.015^i–k^	873.60 ± 30.16^f–k^
Kalshipa	118.35 ± 0.02^a–g^	33.72 ± 1.06^a,b^	24.76 ± 9.38^e,f^	15.35 ± 3.46^f^	10.70 ± 1.08^d–f^	3.92 ± 4.83^a,b^	4.30 ± 4.66^a–e^	5.83 ± 3.62^b–d^	1.70 ± 0.01^f^	2.25 ± 0.028^b^	0.87 ± 0.003^b–d^	819.01 ± 86.87^f–l^
Rangakomal	126.16 ± 13.53^a–c^	24.48 ± 0.91^b–h^	30.32 ± 15.62^b–f^	19.30 ± 4.57^b–f^	13.55 ± 0.44^a–f^	2.87 ± 3.72^a,b^	4.58 ± 4.64^a–e^	6.10 ± 3.91^a–d^	1.28 ± 0.01^o,p^	1.54 ± 0.010^g^	0.51 ± 0.011^g,h^	758.32 ± 59.97^h–l^
Sadanunia	111.87 ± 2.91^c–g^	26.65 ± 3.41^b–g^	32.72 ± 0.02^a–f^	21.60 ± 0.09^a–f^	16.32 ± 2.28^a,b^	2.78 ± 3.37^a,b^	4.32 ± 4.54^a–e^	5.07 ± 3.62^b–d^	1.00 ± 0^w^	1.16 ± 0.003^s,t^	0.62 ± 0.014f.^-g^	421.69 ± 83.96^n^
Tulsibhog	129.02 ± 6.52^a,b^	26.22 ± 6.34^b–g^	24.83 ± 8.15^e,f^	18.03 ± 3.81^b–f^	12.73 ± 0.56^a–f^	2.65 ± 3.27^a,b^	4.67 ± 5.18^a–e^	6.02 ± 4.36^a–d^	1.61 ± 0.01^g,h^	1.43 ± 0.009^h,i^	0.39 ± 0.009^h–j^	644.19 ± 16.41^k–n^
Kalturey	113.10 ± 7.73^b–g^	28.42 ± 0.63^a–f^	24.07 ± 9.23^f^	20.67 ± 1.79^b–f^	13.67 ± 1.74^a–f^	4.63 ± 6.31^a^	6.03 ± 6.74^a,b^	7.50 ± 6.59^a–d^	1.07 ± 0.01^v^	1.46 ± 0.010^h^	0.26 ± 0.006^k,l^	880.35 ± 45.99^e–k^
Kataribhog	103.94 ± 9.08^g,h^	31.07 ± 1.79^a–d^	24.10 ± 9.23^e,f^	16.45 ± 1.39^e,f^	11.38 ± 4.69^c–f^	3.38 ± 4.64^a,b^	4.23 ± 5.23^a–e^	6.07 ± 3.91^a–d^	1.30 ± 0.01^n,o^	1.10 ± 0.002^u^	0.07 ± 0.001^n^	909.42 ± 10.24^d–k^
A-1-1	111.80 ± 8.62^c–g^	28.15 ± 6.10^a–f^	27.45 ± 18.03^c–f^	20.80 ± 9.66^b–f^	14.18 ± 1.48^a–e^	2.42 ± 3.18^a,b^	4.05 ± 3.41^a–e^	5.03 ± 3.48^b–d^	1.26 ± 0.01^p,q^	1.52 ± 0.010^g^	0.43 ± 0.009^h,i^	805.91 ± 22.70^d–k^
Dubarikomal	114.10 ± 14.38^a–g^	23.53 ± 0.51^b–h^	27.10 ± 6.36^c–f^	17.90 ± 0.70^d–f^	11.72 ± 2.75^b–f^	4.02 ± 4.83^a,b^	6.10 ± 6.17^a,b^	8.77 ± 6.45^a–c^	1.21 ± 0.01^s,t^	1.13 ± 0.003^t,u^	0.23 ± 0.005^k,m^	1036.15 ± 292.18^g–l^
Darmaphou	117.12 ± 9.86^a–g^	24.18 ± 3.74^b–h^	28.98 ± 11.14^b–f^	20.95 ± 4.83^b–f^	12.92 ± 0.11^a–f^	2.02 ± 2.56^a,b^	3.60 ± 4.00^b–e^	6.35 ± 5.06^a–d^	1.57 ± 0.01^i,j^	1.56 ± 0.030^g^	0.82 ± 0.019^d,e^	821.48 ± 47.51^a–h^
Dudheswar	122.71 ± 7.66^a–e^	20.55 ± 1.06^e–h^	24.80 ± 10.13^e,f^	17.73 ± 1.55^b–f^	12.03 ± 1.13^b–f^	2.77 ± 3.67^a,b^	4.15 ± 4.73^b–e^	6.57 ± 4.57^a–d^	1.80 ± 0.01^d^	1.92 ± 0.021^d^	0.98 ± 0.022^a,b^	1019.68 ± 73.94^f–l^
Dudhkalam	110.86 ± 6.93^c–g^	21.47 ± 2.07^c–h^	26.22 ± 14.54^d–f^	19.10 ± 4.85^b–f^	12.22 ± 1.48^a–f^	3.45 ± 4.64^a,b^	4.95 ± 5.82^a–e^	7.60 ± 5.37^a–d^	1.20 ± 0.004^t^	1.26 ± 0.006^o,p^	0.13 ± 0.003^m,n^	790.76 ± 1.28^b–h^
Ranpha	110.03 ± 5.70^c–g^	25.08 ± 1.57^b–h^	24.63 ± 9.28^e,f^	16.50 ± 1.55^e,f^	11.70 ± 1.64^b–f^	3.83 ± 4.66^a,b^	6.48 ± 4.92^a–d^	9.02 ± 4.54^a–c^	1.36 ± 0.008^m^	1.21 ± 0.018^q,r^	0.20 ± 0.004^l,m^	1159.83 ± 317.10^g–l^
Gobindabhog	114.28 ± 9.55^a–g^	29.30 ± 2.21^a–e^	29.35 ± 11.90^b–f^	20.88 ± 3.13^b–f^	12.52 ± 0.63^a–f^	5.40 ± 7.35^a^	6.28 ± 7.33^a–d^	8.67 ± 7.91^a–c^	1.23 ± 0.005^r,s^	1.31 ± 0.015^m,n^	0.49 ± 0.011^g,h^	1309.86 ± 38.54^a–e^
Konkanijoha	115.44 ± 7.48^a–g^	28.10 ± 2.020^a–f^	24.12 ± 5.82^e,f^	18.37 ± 1.08^c–f^	11.28 ± 1.90^d–f^	2.87 ± 3.01^a,b^	4.00 ± 3.53^a–e^	6.30 ± 3.62^a–d^	1.57 ± 0.009^i,j^	1.41 ± 0.013^i,j^	0.72 ± 0.016^e,f^	1194.01 ± 27.94^a^
Kalokhasa	120.87 ± 11.39^a–f^	29.58 ± 4.12^a–e^	28.52 ± 12.42^b–f^	19.32 ± 2.80^b–f^	12.63 ± 2.12^a–f^	2.40 ± 3.25^a,b^	3.38 ± 3.51^c–e^	4.48 ± 3.27^c,d^	1.07 ± 0.001^v^	1.28 ± 0.016^n,o^	0.16 ± 0.003^l–n^	971.10 ± 71.61^c,i^
Kabra	118.70 ± 10.78^a–g^	31.82 ± 1.39^a–c^	29.67 ± 5.65^b–f^	21.10 ± 0.14^b–f^	11.68 ± 1.67^b–f^	2.93 ± 4.05^a,b^	4.32 ± 5.35^a–e^	7.25 ± 7.00^a–d^	1.25 ± 0.01^q,r^	1.31 ± 0.015^m,n^	0.16 ± 0.003^k–n^	792.49 ± 6.52^g–l^
Radhunipagol	119.09 ± 8.50^a–g^	25.40 ± 1.50^b–h^	30.23 ± 10.32^b–f^	22.92 ± 4.40^a–f^	11.38 ± 0.73^c–f^	2.42 ± 3.04^a,b^	4.30 ± 4.76^a–e^	7.10 ± 5.98^a–d^	1.20 ± 0.004^t^	1.34 ± 0.015^k–m^	0.61 ± 0.009^f,g^	960.64 ± 205.6^c–j^
Kalojeera	111.95 ± 7.84^c–g^	26.37 ± 7.63^b–g^	31.52 ± 15.53^a–f^	20.35 ± 1.72^b–f^	11.35 ± 1.43^d–f^	3.52 ± 4.50^a,b^	5.32 ± 5.06^a–e^	8.12 ± 5.77^a–c^	1.10 ± 0.002^u^	1.33 ± 0.038^l,m^	0.28 ± 0.016^j–l^	1096.76 ± 88.62^a–f^
Chakhao sel-1	113.99 ± 19.94^a–g^	31.48 ± 7.00^a–d^	34.12 ± 16.14^a–f^	22.22 ± 5.86^a–f^	12.90 ± 1.17^a–f^	5.50 ± 7.40^a^	6.30 ± 7.21^a–d^	9.25 ± 9.12^a–c^	1.74 ± 0.006^e^	1.82 ± 0.019^e^	0.97 ± 0.0007^a–c^	980.15 ± 84.42^c–i^
Chakhao Poiterein	126.48 ± 21.99^a–c^	16.37 ± 2.26^g,h^	35.20 ± 14.56^a–f^	25.20 ± 7.44^a,b^	13.65 ± 2.09^a–f^	4.17 ± 5.70^a,b^	4.95 ± 6.38^a–e^	6.75 ± 6.24^a–d^	1.46 ± 0.01^k^	1.25 ± 0.010^o–q^	0.51 ± 0.011^g,h^	728.92 ± 190.5^i–m^
Chapka Chakhao	116.57 ± 7.21^a–g^	15.37 ± 1.79^h^	36.37 ± 0.23^a–d^	23.40 ± 4.43^a–e^	13.63 ± 0.56^a–f^	3.10 ± 4.33^a,b^	4.50 ± 4.76^a–e^	6.92 ± 7.18^a–d^	1.97 ± 0.0007^a^	2.38 ± 0.031^a^	1.00 ± 0^a^	864.71 ± 6.87^f–k^
Chakhao sel-2	129.98 ± 16.52^a^	22.45 ± 7.18^c–h^	36.88 ± 10.44^a–d^	29.30 ± 6.22^a^	16.02 ± 3.79^a–c^	2.77 ± 3.81^a,b^	3.90 ± 4.43^a–e^	5.52 ± 5.35^b–d^	1.10 ± 0.002^u^	1.31 ± 0.015^m,n^	0.67 ± 0.007^f^	776.27 ± 257.47^h–l^
Chakhao sel-3	124.87 ± 17.52^a–d^	20.25 ± 2.61^e–h^	39.63 ± 11.17^a,b^	26.72 ± 3.60^a,b^	12.62 ± 0.73^a–f^	3.93 ± 5.56^a,b^	4.85 ± 5.86^a–e^	6.25 ± 6.52^a–d^	1.26 ± 0.006^p,q^	1.36 ± 0.038^k,l^	0.21 ± 0.018^k–m^	700.35 ± 139.86^i–m^
Kalonunia	104.49 ± 1.32^f–h^	25.18 ± 8.17^b–h^	26.08 ± 5.63^d–f^	19.37 ± 1.22^b–f^	11.85 ± 3.27^b–f^	4.27 ± 5.75^a,b^	6.00 ± 7.30^a–d^	8.33 ± 7.87^a–c^	1.56 ± 0.01^j^	1.34 ± 0.015^k–m^	0.80 ± 0.004^d,e^	1169.96 ± 26.08^a–d^
KNS-2′-1	108.69 ± 3.64^d–g^	16.77 ± 2.30^g,h^	32.28 ± 13.22^a–f^	23.20 ± 4.19^a–f^	12.15 ± 1.57^a–f^	4.07 ± 5.27^a,b^	6.48 ± 6.81^a–d^	8.65 ± 7.14^a–c^	1.39 ± 0.009^l^	1.23 ± 0.005^p–r^	0.61 ± 0.009^f,g^	1076.83 ± 57.41^a–g^
KNS-3′-1 Uttar Sugandhi (IET 24,616)	107.82 ± 2.29^e–g^	22.05 ± 3.74^c–h^	30.17 ± 10.98^b–f^	19.60 ± 0.61^b–f^	12.77 ± 1.93^a–f^	5.18 ± 6.67^a^	7.58 ± 6.85^a,b^	9.40 ± 7.87^a–c^	1.36 ± 0.008^m^	1.28 ± 0.016^n,o^	0.67 ± 0.007f.	1265.32 ± 31.90^a,b^
KNS-2-1-1	116.51 ± 5.12^a–g^	18.10 ± 1.08^f–h^	32.70 ± 11.64^a–f^	21.82 ± 0.44^a–f^	11.17 ± 1.08^b–f^	5.70 ± 7.91^a^	8.02 ± 9.07^a^	10.65 ± 9.73^a,b^	1.59 ± 0.01^h,i^	1.21 ± 0.018^q,r^	0.66 ± 0.015f.	914.44 ± 141.49^d–k^
KNS-2B-S1	111.92 ± 3.50^c–g^	20.40 ± 1.08^e–h^	35.27 ± 10.41^a–e^	23.00 ± 0.28^a–f^	11.68 ± 1.34^d–f^	4.12 ± 5.49^a,b^	5.70 ± 6.45^a–d^	9.55 ± 7.14^a–c^	1.62 ± 0.01^g^	1.38 ± 0.014^j,k^	0.66 ± 0.015^f^	1229.66 ± 115.87^a–c^
Chakhao Sampark	118.57 ± 0.34^a–g^	21.43 ± 13.52^c–h^	33.10 ± 3.58^a–f^	24.72 ± 1.76^a,b^	14.57 ± 1.41^a,b^	0.50 ± 0.70^b^	1.33 ± 1.17^e^	1.93 ± 0.09^b^	1.10 ± 0.002^u^	1.20 ± 0.004^r,s^	0.43 ± 0.329^h,i^	469.20 ± 43.55^m,n^
TSPM-3-1 TPUR-B-1(IET 28,104)	92.12 ± 5.21^h–i^	20.62 ± 0.73^e–h^	37.85 ± 6.85^a–c^	19.62 ± 5.11^b–f^	13.75 ± 1.62^a–f^	1.87 ± 2.63^a,b^	5.43 ± 6.83^a–e^	4.82 ± 4.40^c,d^	1.89 ± 0.02^b^	2.18 ± 0.027^c^	0.85 ± 0.019^c,d^	676.96 ± 52.05^j–n^
T4M-3-5	85.80 ± 0.28^i^	17.15 ± 0.54^g,h^	35.17 ± 0.80^a–f^	29.05 ± 3.88^a^	16.72 ± 3.46^a^	3.18 ± 4.31^a,b^	2.75 ± 3.08^d,e^	5.23 ± 5.79^b–d^	1.85 ± 0.01^c^	1.36 ± 0.008^k,l^	0.61 ± 0.009f.^-g^	558.63 ± 40.06^l–n^
T6M-3-3	80.40 ± 1.45^i^	21.22 ± 5.53^d–h^	34.48 ± 5.25^a–f^	26.08 ± 0.91^a–c^	14.90 ± 1.13^a–d^	3.60 ± 4.99^a,b^	4.28 ± 4.97^a–e^	7.08 ± 7.33^a–d^	1.98 ± 0.02^a^	1.67 ± 0.007f.	0.61 ± 0.009^f–g^	783.51 ± 129.38^h–l^
TSP6-M3-4	81.83 ± 1.17^i^	21.73 ± 0.04^c–h^	34.60 ± 10.13^a–f^	26.65 ± 3.46^a,b^	13.77 ± 1.64^a–f^	3.10 ± 4.19^a,b^	4.57 ± 5.70^a–e^	6.50 ± 6.92^a–d^	1.98 ± 0.02^a^	1.80 ± 0.004^e^	0.90 ± 0.002^a–d^	642.29 ± 19.09^k–n^
CV^##^	4.25	12.07	10.40	10.64	10.47	31.86	23.97	23.61	0.59	0.89	7.52	9.46
F value	**	**	–	*	–	–	–	–	**	**	**	**

CV means Coefficient of Variance, Statistical significance was measured by F test, **means values are statistically significant at p < 0.01.

### Identification of important phenotypic characters related to blast disease

**Figure 6:**
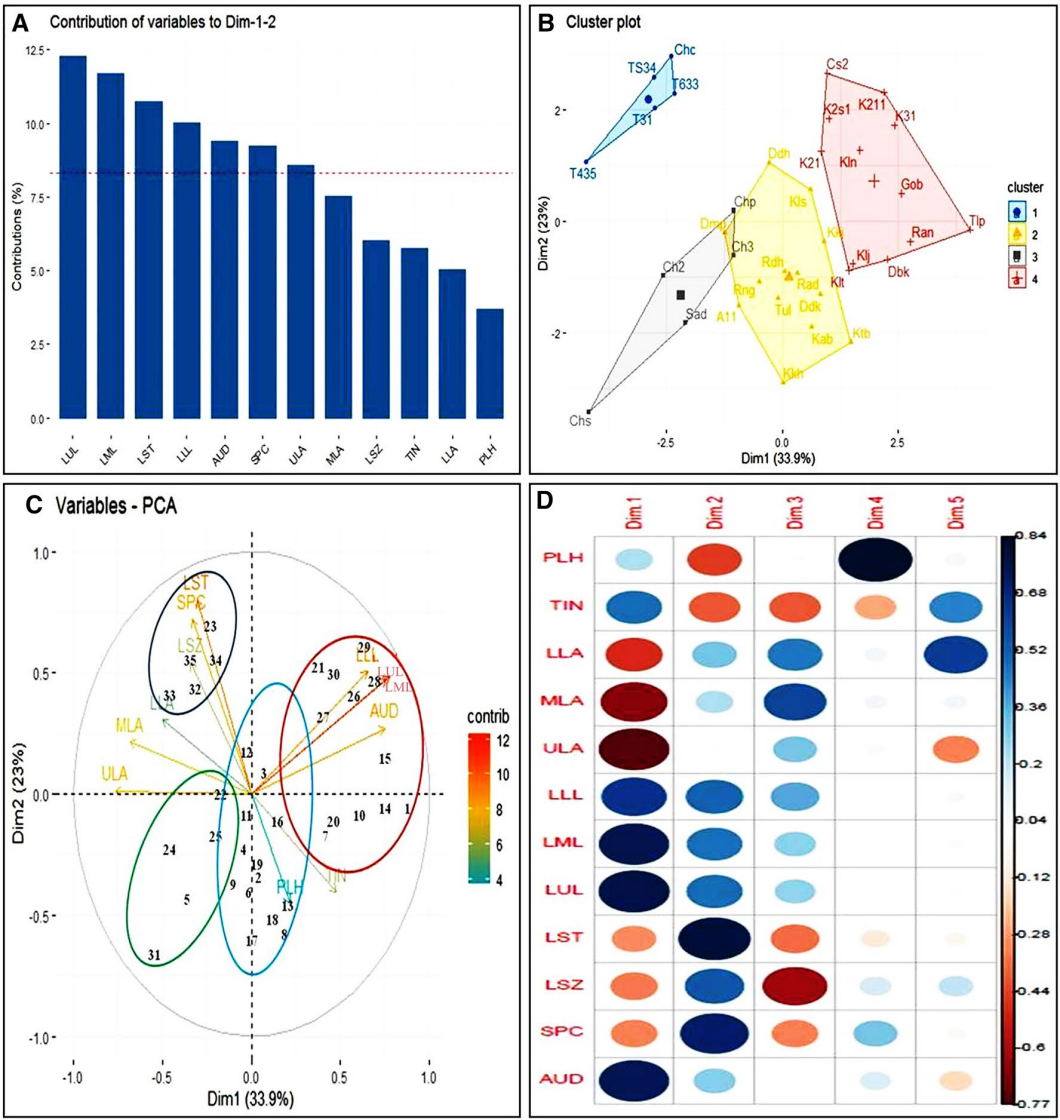
PCoA biplot of thirty-five genotypes based on their important phenotypic and disease related attributes. All the analysis was performed using R software^31^, version 3.5.1, Patched (2018-07-02 r74950) Platform: x86_64-w64mingw32/x64 (64-bit) (https://www.R-project.org/). (**A**) Contribution of various phenotypic and disease related attributes based on the mean values for the grouping of the rice genotypes. The Y-axis depicts the contribution of the parameters in percentage (%). (**B**) Cluster analysis of the rice genotypes, the X and Y axis represent the PC1 (dim1) and PC2 (dim2) and the percentage (%) of total variation defined by PC1 and PC2. (**C**) PCoA biplot exhibiting the grouping of the genotypes as well as the phenotypic and disease related attributes. The X and Y axis represent the PC1 (dim1) and PC2 (dim2) and the percentage (%) of total variation defined by PC1 and PC2. (**D**) Dimension wise distribution of the phenotypic and disease related attributes contributing towards the clustering of the genotypes. The genotypes are designated either numerically or in short form as following 1. ‘Tulaipanji’ (Tlp), 2. ‘Radhatilak’ (Rad), 3. ‘Kalshipa’ (Kls), 4. ‘Rangakomal’ (Rng), 5. ‘Sadanunia’ (Sad), 6. ‘Tulsibhog’ (Tul), 7. ‘Kalturey’ (Klt), 8. ‘Kataribhog’ (Ktb), 9. ‘A-1-1’ (A11), 10. ‘Dubarikomal’ (Dbk), 11. ‘Dharmaphou’ (Dmp), 12. ‘Dudheswar’ (Ddh), 13. ‘Dudhkalam’ (Ddk), 14. ‘Rampha’ (Ran), 15. ‘Gobindabhog’ (Gob), 16. Konkanijhoha (Kkj), 17. Kalokhasa (Kkh), 18. Kabra (Kab), 19. Radhunipagol (Rdh), 20. Kalojeera (Klj), 21. Chakhao Sel.-1 (Cs1), 22. Chakhao Poiterin (Chp), 23. Chapka Chakhao (Chc), 24. Chakhao sel-2 (Ch2), 25. Chakhao sel-3 (Ch3), 26. Kalonunia (Kln), 27. KNS-2′-1 (K2′1), 28. KNS-3′-1 (K3′1), 29. KNS-2-1-1 (K211), 30. KNS-2B-S1 (K2s1), 31. Chakhao Sampark (Chs), 32. TPUR-B-1(TSPM-3–1) (T31), 33. T4M-3-5 (T435), 34. T6M-3-3 (T633), 35. TSP6-M3-4 (TS34). The phenotypic attributes are denoted as *PLH *plant height, *TIN *Tiller Number, *LLA *lower leaf angle, *MLA *middle leaf angle, *ULA *upper leaf angle, *LLL *lesion no. In Lower Leaf, *LML *lesion no. In Middle Leaf, *LUL *lesion no. In Upper Leaf, *LST *lesion type, *LSZ *lesion size, *SPC *sporulation centre, *AUD *AUDPC.

Phenotypic data like plant height (PLH), tiller number (TLN), upper, lower and middle leaf angles (ULA, MLA, LLA respectively) in selected rice genotypes and their impact on the disease parameters like lesion numbers in upper, middle and lower leaves (LUL, LML, LLL respectively), lesion size (LSZ), sporulation center (SPC) and AUDPC were calculated (Table 6). Principal Component analysis showed that the disease parameters like lesion number in upper middle and lower leaf (LUL, LML and LLL), lesion type (LST), sporulation center (SPC) and AUDPC were found to be significantly contributing to the clustering of the genotypes (Fig. 6A, Supplementary information 3b). Among the morphological traits only upper leaf angle (ULA) significantly contributed to the clustering of the genotypes. Cluster analysis based on these disease parameters have resulted in four distinguished clusters for these genotypes (Fig. 6B). Cluster I and III constitutes of the genotypes like ‘Sadanunia’, ‘Chakhao Sampark’, ‘TSP6-M3-4’, ‘T4M-3-5’ etc. which showed low AUDPC value (< 600) with a smaller number of disease lesions in leaves and considered highly tolerant to the blast disease whereas genotypes in cluster III exhibited relatively higher AUDPC and is considered to be less tolerant to the disease. Cluster IV represented the cultivars which has high AUDPC (> 1000) with dense disease lesion in all leaves and is considered to be highly susceptible to the blast disease. PCA analysis has demonstrated that AUDPC and disease lesions on lower, upper and middle leaf are placed on the same dimension whereas the leaf angle parameters were found to be falling in the opposite dimension (Fig. 6C). Correlation and regression analysis suggested that upper and middle leaf angle exhibits a significant negative correlation with AUDPC (Table 7, Fig. 6D). Tiller numbers and plant height were found to have non-significant but positive correlation with occurrence of the disease. The correlation study between the nutritional and disease parameters were also performed. Aroma (ARO) and antioxidant activity (ANT) was found to be have significant but weak positive correlation with AUDPC whereas Non-Reducing Sugar (NRS) content was found to have very weak negative correlation with AUDPC (Supplementary information 4) suggesting that the nutritional traits and disease resistance are unrelated independent characters.

**Table 7:** Correlation and regression analysis of the morphological and disease related characters with AUDPC.

Correlations												
	Plh	Tin	Lla	Mla	Ula	Lll	Lml	Lul	Lst	Lss	Spc	AUDPC
**AUDPC**												
Pearson correlation	0.078	0.145	− 0.183	− 0.261*	− 0.348**	0.124	0.149	0.236*	− 0.040	− 0.123	0.014	1
Sig. (2-tailed)	0.521	0.232	0.130	0.029	0.003	0.306	0.220	0.049	0.743	0.309	0.909	

*Correlation is significant at the 0.05 level (2-tailed). **Correlation is significant at the 0.01 level (2-tailed).

*PLH* plant height, *TLN* Tiller number, *LLA* lower leaf angle, *MLA* middle leaf angle, *ULA* upper leaf angle, *LLL* lesion number in lower leaf, *LML* lesion number in middle leaf, *LUL* lesion number in upper leaf, *LST* lesion type, *LSS* lesion size, *Spc* sporulation center.

### Assessment of *pi* genes with the disease

**Figure 7:**
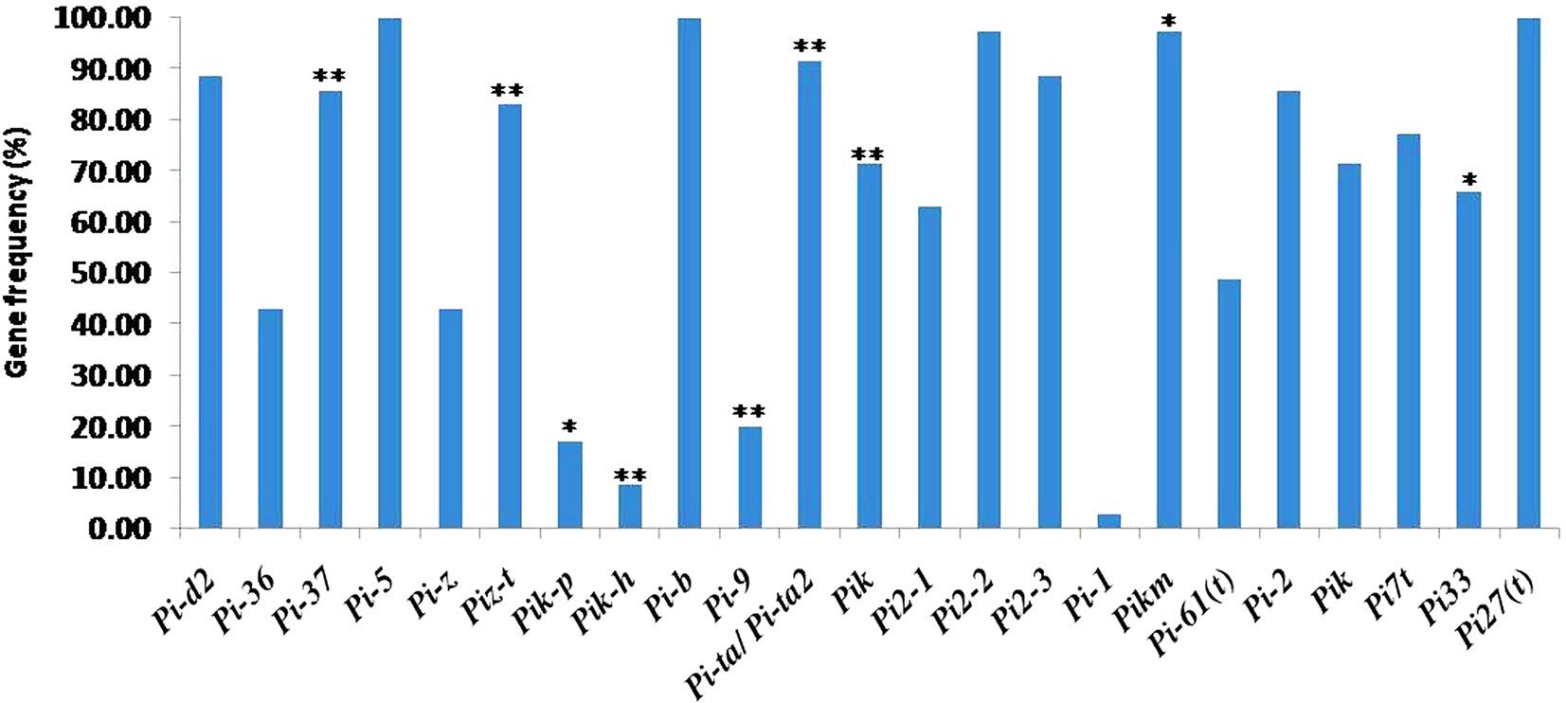
Distribution of twenty-three major blast resistant genes in the local landraces. Frequency of the genes in the landraces is calculated. Significant Kendall’s-tau b association of the Pi gene with tolerance to the disease is represented by ‘*’ (**mean level of significance < 0.01, *means level of significance < 0.05). Kendall's tau-b correlation coefficient was calculated using IBM-SPSS trial version 20^33^ (https://www.ibm.com/analytics/spss-statistics-software).

The selected genotypes were screened for the presence of twenty-three well characterized rice blast resistant *pi* gene (Table 8). Almost all the landraces were found to possess number of *pi* gene. Genotypes like ‘A-1-1’ and ‘Kataribhog’ contain all the twenty-three *pi* genes (Supplementary information 5). Lowest number of *pi* genes was found in ‘T6M-3-3’ and ‘T4M-3-5’. *Pi* 27t showed the maximum density in the population and was found in every genotype. Similarly, *Pi5, Pizt, pib, Pikm and Pita/Pita2* was found in these landraces with high density (Fig. 7). *Pik-h, pi-9, pi-1* were found with very low frequency in these genotypes. To address the association of the *pi* genes, present in these genotypes with occurrence of the disease Kendall’s-tau association analysis was performed using R package. All the genotypes from the cluster one, two and some genotypes from cluster three with less than 800 AUDPC was considered as tolerant and genotypes falling in cluster three and four with more than 800 AUDPC were considered as susceptible. Six *pi* genes *pi37, pizt, pikh, pi9, pita-pita2,* and *pik* showed strong Kendal-tau b association (< 0.001) with blast tolerance in these genotypes (Fig. 7, Supplementary information 6). *Pikp, pikm and pi-33* also exhibited significant correlation with blast tolerance, whereas fourteen out of twenty-three markers were found to have no association with blast tolerance in these genotypes (Fig. 7).

**Table 8:** Details of *Pi* genes analysed in the study.

Sl. no.	Gene name	Forward sequence	Reverse sequence	Chromosome no.	Amplicon size (bp)	Annealing temp (°C)	References
1.	Pi-d2	TTGGCTATCATAGGCGTCC	ATTTGAAGGCGTTTGCGTAGA	6	1057	55	^63^
2.	Pi-36	CAATGTGTGACTTGTGCGGACT	TCTTCCATCTCGGATTTCGTGT	8	1036	55	^64^
3.	Pi-37	TCTTGAGGGTCCCAGTGTAC	CGAACAGTGGCTGGTATCTC	1	1149	55	^65^
4.	Pi5	TCCTCCTCTTCGGACACCTC	CGGACGAGCGATAGTGATCC	9	594	55	^65^
5.	Pi-z	GGACCCGCGTTTTCCACGTGTAA	AGGAATCTATTGCTAAGCATGAC	6	292	60	^66^
6.	Piz-t	TTGCTGAGCCATTGTTAAACA	ATCTCTTCATATATATGAAGGCCAC	6	257	56	^67^
7.	Pik-p	ATAGTTGAATGTATGGAATGGAAT	CTGCGCCAAGCAATAAAGTC	11	148	60	^67^
8.	Pik-h	CATGAGTTCCATTTACTATTCCTC	ACATTGGTAGTAGTGCAATGTCA	11	1500	55	^68^
9.	Pi-b	GACTCGGTCGACCAATTCGCC	ATCAGGCCAGGCCAGATTTG	2	388	60	^67^
10.	Pi-9	ATGGTCCTTTATCTTTATTG	TTGCTCCATCTCCTCTGTT	6	2000	53	^61^
11.	Pi-ta/Pi-ta2	AGCAGGTTATAAGCTAGGCC	CTACCAACAAGTTCATCAAA	12	1042	58	^69^
12.	Pik	GCCACATCAATGGCTACAACGTT	CCAGAATTTACAGGCTCTGG	11	112	60	^67^
13.	Pi2-1	GATTTAGTTCAGGAAAACACTC	TGGAAGCCTCATTGATCATC	12	2344	55	^70^
14.	Pi2-2	CGTTGTATAGGACAGTTTCATT	AATCTAGGCACTCAAGTGTTC	6	436	50	^71^
15.	Pi2-3	CAGCGATGGTATGAGCACAA	CGTTCCTATACTGCCACATCG	5	450	57	^72^
16.	Pi-1	GTGTAAATCATGGGCACGTG	AGATTGGCTCCTGAAGAAGG	11	170	55	^73^
17.	Pik-m	CGTGCTGTCGCCTGAATCTG	CACGAACAAGAGTGTGTCGG	11	619	55	^74^
18.	Pi-61(t)	AGATGATAAGCTTGCGGACC	ATGCAGATGAGTCCCTCCAC	11	210	55	^75^
19.	Pi-2	CTCCTTCAGCTGCTCCTC	TGATGACTTCCAAACGGTAG	6	200	58	^76^
20.	Pik	CGTGCTGTCGCCTGAATCTG	CACGAACAAGAGTGTGTCGG	11	150	58	^76^
21.	Pi7t	CACTCACACGAACGACTGAC	CGCAGGTTCTTGTGAAATGT	11	200	56	^76^
22.	Pi-33	Motif = (TAT)5C(ATT)15		8	166	56	^77^
23.	Pi-27(t)	Motif = (CT)17		1	162	56	^76^

### Fitted model for prediction of susceptibility to blast disease based on morphological traits

Based on the results of Principal Component analysis, significantly contributing variables except AUDPC were chosen for development of a logistic regression model for prediction of disease susceptibility of a genotype based on its morphological data. For testing the goodness of fit of the model Hosmer and Lemeshow test was performed which was found non-significant (p value 0.694) for the model at 5% level of significance indicating that the model fits well as for any goodness of fit.

The fitted model is:

$$P\left[ {Blast = \,1} \right] = \frac{1}{{1 + \exp \left( {5.566 + 0.892*LUL - 0.759*LML - 2.845*LST - 0.144*LLL + 2.847*SPC - 0.329*ULA} \right)}}$$

The model predicts that one unit increase in upper leaf lesion (LUL) will increase the odds in favor of blast occurrence by 2.439 times (Table 9, Supplementary information 7). On the other hand, one unit decrease in upper leaf angle (ULA) will increase the odds in favor of blast occurrence by 0.720 times. Sporulation center (SPC) was found to be the most important parameter for the prediction of blast occurrence. The fitted model is highly accurate in the sense that based on significant phenotypic parameters considered for the purpose the overall correct classification rate among all the thirty-five genotypes is 75.7% whereas the correct classification rate is 86.7% within the susceptible genotypes under consideration (Supplementary information 7).

**Table 9:** The logistic regression model was fitted by considering only significant variables except AUDPC based on PCA results.

Parameters	B	S.E.	Sig	Exp(B)
Lesion number in upper leaf (LUL)	0.892	0.332	0.007	2.439
Lesion number in middle leaf (LML)	− 0.759	0.463	0.049	0.468
Lesion type (LST)	− 2.845	1.467	0.061	0.058
Lesion number in lower leaf (LLL)	− 0.144	0.421	0.070	0.866
Sporulation center (SPC)	2.847	1.526	0.057	17.244
Upper leaf angle (ULA)	− 0.329	0.149	0.027	0.720
Constant	5.566	2.776	0.045	261.457

Where B signifies coefficient for fitted logistic regression model, SE means standard error of the parameter estimate, Sig. indicates P values and EXP(B) signifies odd in favorable Blast Occurrence. Here, based on AUDPC values, disease occurrence has been calculated as 1 when AUDPC > 800.

Rice blast caused by fungal pathogen *Magnaporthe oryzae* has been major constrain causing huge yield losses every year and considered as one of the most destructive disease of rice^50–53^. Indian subcontinent has faced seven severe epidemics due to the disease in last two decades^53^. Rice blast is influenced by several climatic conditions as relative humidity; temperature, light intensity etc. Based on the weather parameters several disease prediction models are available in different countries^50,54^, but disease prediction model based on morphological traits of the plant have been linked with blast disease is scarce. Morphological traits, like plant height, tiller number; leaf angle etc. has been reported to impact disease severity in many crops. Plant height and percentage of unfilled grain has been reported to be positively correlated to the severity of the disease^55^. Much earlier Mohanty et al*.*^56^ has reported a positive correlation of leaf angle, leaf pubescence, epicuticular wax, and quantity of deposition of conidia with disease incidence. The cultivars in present study have shown a range of adaptability to the blast disease. Our data suggest a significant negative correlation of leaf angle of the genotypes with occurrence of the disease. The leaves with smaller leaf angles may protect the spores from direct sunlight and favors the spore germinations^50,57^ as direct sunlight has very detrimental effect to the germination of blast spore^58^. More over reduced leaf angles result in dense canopy cover and increases canopy temperature which may lead congenial micro-environment for the fungus and may positively influence the selective outbreak of the disease in these genotypes.

Search for resistant sources against blast disease has been going on all over the world and around 120 resistant genes have been reported till date. Majority of the *pi* genes are known to encode nucleotide binding site (NBS)-leucine rich repeats (LRR) proteins^59^. The genes which were found to be associated with blast tolerance of the genotypes in the present study, like *Pi 9* and *pi 37* etc. are known to confer broad spectrum resistance to blast disease^60,61^. *Pikh, pikm* and *pikp* are known to be different allele of *pik* which act as a two-protein system in the plant against blast^62^. *Pita* and *pizt* are also known to exhibit complete resistance to blast disease. It can be presumed that the landraces have acquired this resistant gene while combating continuous pathogen pressure for a long period of time. Along with these known genes the presence of unknown genes may also be contributing to the resistance of the genotypes against the blast disease. Detail investigation of the resistant genotypes may lead to the isolation of novel genes or QTLs linked with blast disease resistance.

## Conclusion

In the current study, proximate analysis of non-Basmati aromatic rice genotypes has been performed which has successfully zeroed on cultivars like, ‘Kataribhog’, ‘Sadanunia’, ‘Kalshipa’ etc. having low glycemic index, high resistant starch and high antioxidant potential respectively. On the basis of collective evidence from two years field trial and in vitro experiments blast resistant local genotypes like ‘Sadanunia’, ‘Chakhao Sampark’, ‘T4M-3-5’ etc. were identified. Allele mining for the resistant genes in these genotypes demonstrated significant association of six *pi* genes with resistance against blast disease. The prediction model with plant morphological characters were developed with an accuracy level of more than 85% for the occurrence of blast disease. The scope for pushing non-Basmati scented rice in the domestic as well as the global market is growing and it is high time to highlight and popularize these folk cultivars for their nutritional and disease resistance attributes. Many of these traditional cultivars may also be used as donors for traits like biotic, abiotic stress resistance and for aroma in rice improvement programs. In addition, the use of these cultivars as donor will result in large number of segregants in subsequent generation due to the wide genetic base of these cultivars. It is also important to build strategies for improvement of these genotypes in terms of yield, photosensitivity, disease resistance, cooking quality, and benefit to human health etc. using mutation breeding or biotechnological tools keeping the desirable traits like aroma intact. Combined approaches for the betterment of these heirloom rice cultivars will encourage the farmers to take on the cultivation of their own traditional genotypes over the HYVs.

## Supplementary Information

Supplementary Information 1.

Supplementary Information 2.
