# Supplementary material for: Evaluation of indigenous aromatic rice cultivars from sub-Himalayan Terai region of India for nutritional attributes and blast resistance
Source: Sci Rep. 2021 Feb 26;11:4786. doi: 10.1038/s41598-021-83921-7 (PMC7910543; doi:10.1038/s41598-021-83921-7)
Supplement: Supplementary file 1 — Supplementary Information 1. [file 41598_2021_83921_MOESM1_ESM.pdf]

Supplementary Information

Title: Evaluation of indigenous aromatic rice cultivars from sub -Himalayan Terai region of India for nutritional attributes and blast resistance

Debayan Mondal[1], Prudveesh Kantamraju[1], Susmita Jha[2], Gadge Sushant Sundarrao[3], Arpan Bhowmik[4], Hillol Chakdar[5], Somnath Mandal[1], Nandita Sahana[1*] Bidhan Roy[3], Prateek Madhab Bhattacharya[2], Apurba Kr Chowdhury[2], Ashok Choudhury[6]

[1]Department of Biochemistry, Uttar Banga Krishi Viswavidyalaya, Pundibari, Coochbehar-736165

[2]Department of Plant Pathology, Uttar Banga Krishi Viswavidyalaya, Pundibari, Coochbehar-736165

[3]Department of Seed science and Technology, Uttar Banga Krishi Viswavidyalaya, Pundibari, Coochbehar-736165

[4]ICAR-Indian Agricultural Statistics Research Institute, Pusa, New Delhi-110012,

[5]ICAR-National Bureau of Agriculturally important Microorganisms, Mau Nath Bhanjan, UP-275103

[6]Soil Microbiology laboratory, Regional research Station, Uttar Banga Krishi Viswavidyalaya, Pundibari, Coochbehar-736165

Corresponding Authors

nanditasahana@gmail.com (Nandita Sahana)

Supplementary Information 1a: The collected landraces and their place of collection

	Name of land race	Place of collection/Source of the seed
1.	Ayangleima Phou	Central Agriculture University, Imphal, Manipur
2.	Baigonmacchua	Tarai Research Society, Alipurduar, West Bengal
3.	Betho	Alipurduar district, West Bengal
4.	Beto	Tarai Research Society, Alipurduar, West Bengal
5.	Binni	Tarai Research Society, Alipurduar, West Bengal
6.	Birali	Selection UBKV, Pundibari, Cooch Behar, West Bengal
7.	Birali Selection	Selection UBKV, Pundibari, Cooch Behar, West Bengal
8.	Boichi	Alipurduar district, West Bengal
9.	Bonnidhan	Tarai Research Society, Alipurduar, West Bengal
10.	Chakhao Amubi	Central Agriculture University, Imphal, Manipur
11.	Chakhao Angangbi	Central Agriculture University, Imphal, Manipur
12.	Chakhao Poireiton	Central Agriculture University, Imphal, Manipur
13.	Chakhao Sampark	Central Agriculture University, Imphal, Manipur
14.	Chakhao-Selection-1	UBKV, Pundibari, Cooch Behar, West Bengal
15.	Chakhao-Selection-2	UBKV, Pundibari, Cooch Behar, West Bengal
16.	Chakhao-Selection-3	UBKV, Pundibari, Cooch Behar, West Bengal
17.	Chapka Chakhao	Central Agriculture University, Imphal, Manipur
18.	Dharamphou	Central Agriculture University, Imphal, Manipur
19.	Dhyapa	Alipurduar district, West Bengal
20.	Dubarikomal	ICAR-CPRI- Kahikuchi, Kamrup, Assam
21.	Dudhekalam Motajosawa	Tarai Research Society, Alipurduar, West Bengal
22.	Dudhkalam	Tarai Research Society, Alipurduar, West Bengal
23.	Dudhkalam-9	Tarai Research Society, Alipurduar, West Bengal
24.	Dudheswar	Tarai Research Society, Alipurduar, West Bengal
25.	Dudheswar -AD	Tarai Research Society, Alipurduar, West Bengal
26.	Fudugey	Kalimpong, Darjeeling district, West Bengal
27.	Gobindabhog	BCKV, Mohanpur, West Bengal
28.	Jaldhyapa-2	Sitalkuchi, Cooch Behar district, West Bengal
29.	Jaldhyapa-3	Cooch Behar district, West Bengal
30.	Jaldhyapa-AD	Tarai Research Society, Alipurduar, West Bengal
31.	Jasawa-AD	Alipurduar district, West Bengal
32.	Jashoya	Tarai Research Society, Alipurduar, West Bengal
33.	Jhapaka	Kalimpong, Darjeeling district, West Bengal
34.	Jonroi Buna	Uttar Dinajpur district, West Bengal
35.	Kabra	ICAR-CPRI- Kahikuchi, Kamrup, Assam
36.	Kagey Kalimpong	Darjeeling district, West Bengal
37.	Kaike	Tarai Research Society, Alipurduar, West Bengal
38.	Kaloboichi	Tarai Research Society, Alipurduar, West Bengal
39.	Kalodhyapa	Tarai Research Society, Alipurduar, West Bengal
40.	Kalojeera	BCKV, Mohanpur, West Bengal
41.	Kalokhasa	Uttar Dinajpur district, West Bengal
42.	Kalonunia	Cooch Behar district, West Bengal
43.	Kalshipa	Tarai Research Society, Alipurduar, West Bengal
44.	Kalturey	Kalimpong district, West Bengal
45.	Kashiya Binni	Tarai Research Society, Alipurduar, West Bengal
46.	Kataribhog	Alipurduar, West Bengal
47.	Kauka-Selection	UBKV, Pundibari, Cooch Behar, West Bengal

48.	Khaiyamdhan	Tarai Research Society, Alipurduar, West Bengal
49.	Kharadhan PSBSG	Sitalkuchi, Cooch Behar district, West Bengal
50.	Konkonijoha	ICAR-CPRI- Kahikuchi, Kamrup, Assam
51.	Ladu PSBSG	Sitalkuchi, Cooch Behar district, West Bengal
52.	Maitee	Kalimpong, Darjeeling, West Bengal
53.	Malbati	Tarai Research Society, Alipurduar, West Bengal
54.	Malshira	Tarai Research Society, Alipurduar, West Bengal
55.	Mangamuthi	Tarai Research Society, Alipurduar, West Bengal
56.	Pahariboichi	Jalpaiguri district, West Bengal
57.	Pahariboichi-Selection	UBKV, Pundibari, Cooch Behar, West Bengal
58.	Panikuthi Shyamlal	Uttar Dinajpur district, West Bengal
59.	Phoolpakari-1	Tarai Research Society, Alipurduar, West Bengal
60.	Phoolpakari-2	Tarai Research Society, Alipurduar, West Bengal
61.	Phoren Mubi	Central Agriculture University, Imphal, Manipur
62.	Radhatilak PSBSG	Sitalkuchi, Cooch Behar district, West Bengal
63.	Radhatilak-AD	Tarai Research Society, Alipurduar, West Bengal
64.	Radhunipagol	BCKV, Mohanpur, West Bengal
65.	Rampha	ICAR-CPRI- Kahikuchi, Kamrup, Assam
66.	Rongakomal	ICAR-CPRI- Kahikuchi, Kamrup, Assam
67.	Sadamala	Tarai Research Society, Alipurduar, West Bengal
68.	Sadanunia	Cooch Behar district, West Bengal
69.	Sadabhatkalo PSBSG	Sitalkuchi, Cooch Behar district, West Bengal
70.	Satia	Tarai Research Society, Alipurduar, West Bengal
71.	Seshphal	Alipurduar district, West Bengal
72.	Sitalkuchi-1(A-1-1)	UBKV, Pundibari, Coch Behar, West Bengal
73.	Sitalkuchi-2	UBKV, Pundibari, Coch Behar, West Bengal
74.	Sitalkuchi-3	UBKV, Pundibari, Coch Behar, West Bengal
75.	Sitalkuchi-5	UBKV, Pundibari, Coch Behar, West Bengal
76.	Sitalkuchi-6	UBKV, Pundibari, Coch Behar, West Bengal
77.	Tarai Research Society-1	Tarai Research Society, Alipurduar, West Bengal
78.	Tarai Research Society -2	Tarai Research Society, Alipurduar, West Bengal
79.	Tarai Research Society -3	Tarai Research Society, Alipurduar, West Bengal
80.	Tarai Research Society -4	Tarai Research Society, Alipurduar, West Bengal
81.	Tarapakari	Tarai Research Society, Alipurduar, West Bengal
82.	Tarapakari-Selection	UBKV, Pundibari, Coch Behar, West Bengal
83.	Thuri PSBSG	Sitalkuchi, Cooch Behar district, West Bengal
84.	Tulaipanji	Uttar Dinajpur, West Bengal
85.	Tulsibhog	Alipurduar, West Bengal
86.	Tulsimukul	Tarai Research Society, Alipurduar, West Bengal
87.	Uttar Banga Local -3	Tarai Research Society, Alipurduar, West Bengal
88.	Uttar Banga Local -10	Tarai Research Society, Alipurduar, West Bengal
89.	Uttar Banga Local -11	UBKV, Pundibari, Cooch Behar, West Bengal
90.	Uttar Banga Local -13	Tarai Research Society, Alipurduar, West Bengal
91.	Uttar Banga Local -14	Tarai Research Society, Alipurduar, West Bengal
92.	Uttar Banga Local -15	Tarai Research Society, Alipurduar, West Bengal
93.	Uttar Banga Local -17	Tarai Research Society, Alipurduar, West Bengal
94.	Uttar Banga Local -18	Tarai Research Society, Alipurduar, West Bengal
95.	Uttar Banga Local -2 AD	Tarai Research Society, Alipurduar, West Bengal
96.	Uttar Banga Local -3-1	Tarai Research Society, Alipurduar, West Bengal
97.	Uttar Banga Local -5	Tarai Research Society, Alipurduar, West Bengal

98.	Uttar Banga Local -6	Tarai Research Society, Alipurduar, West Bengal
99.	Uttar Banga Local -9	Tarai Research Society, Alipurduar, West Bengal
100.	KNS-2'-1	UBKV, Pundibari, Cooch Behar, West Bengal
101.	KNS-3'-1 (Uttar Sugandhi IET 24616)	UBKV, Pundibari, Cooch Behar, West Bengal
102.	KNS-2-1-1	UBKV, Pundibari, Cooch Behar, West Bengal
103.	KNS-2B-S1	UBKV, Pundibari, Cooch Behar, West Bengal
104.	T4M-3-5	UBKV, Pundibari, Cooch Behar, West Bengal
105.	TSP6-M3-4	UBKV, Pundibari, Cooch Behar, West Bengal
106.	TPUR-B-1 (IET 28104)	UBKV, Pundibari, Cooch Behar, West Bengal
107.	T6M-3-3	UBKV, Pundibari, Cooch Behar, West Bengal

Supplementary Information 1b: Geographical locations of the collection site of the 107 landraces

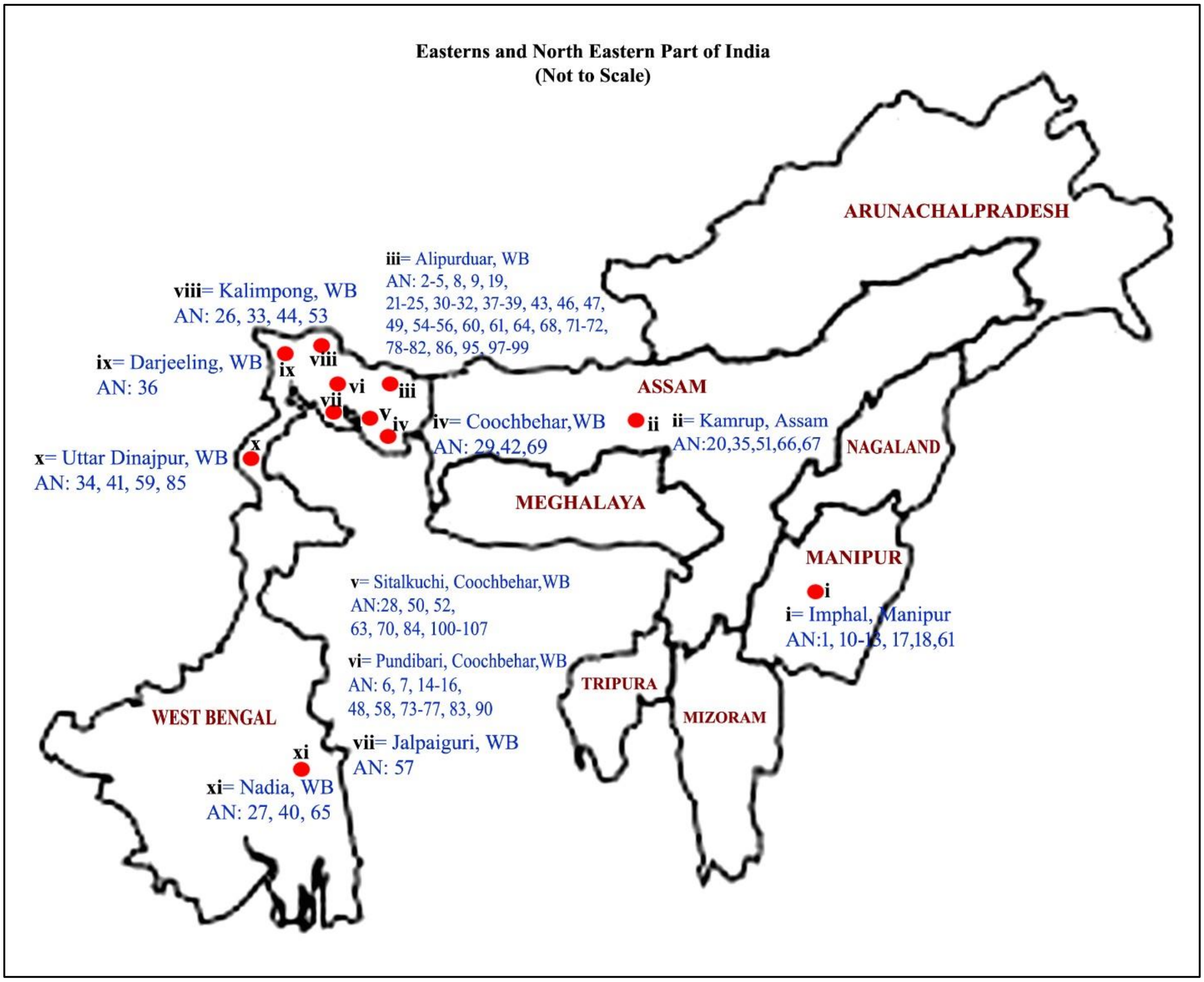

Figure legend 1b: The collection sites for all the genotypes are numbered (i- xi). The genotypes are numbered following supplementary table 1a. The map has been created using Smartdraw software version 7 (URL:https://www.smartdraw.com/). The red dots indicating the sites of collection and the text are added in Microsoft PowerPoint, 2019.

Supplementary Information 2: Dendogram generated separately from SSR, ISSR markers

a) Dendrogram based on SSR markers

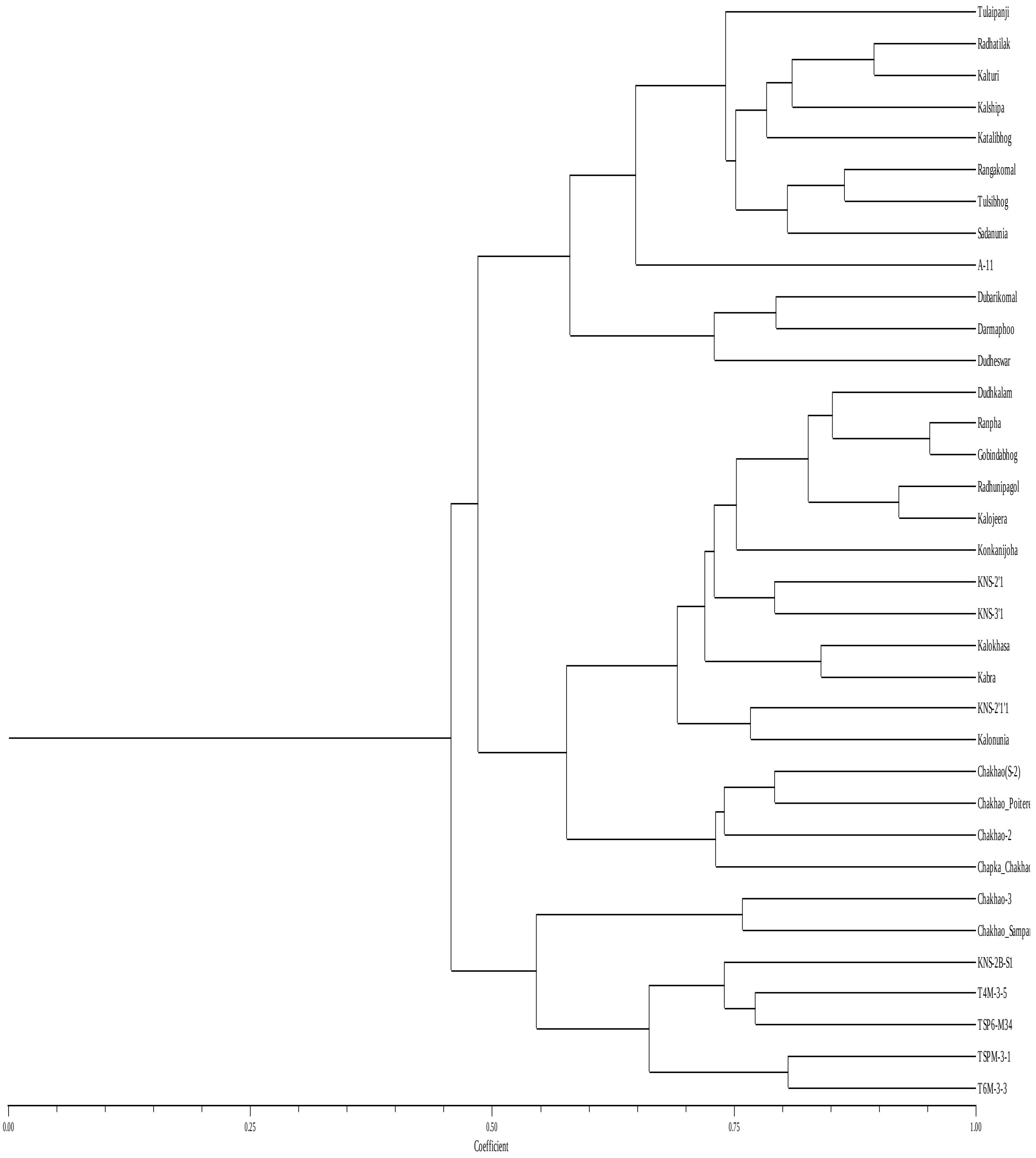

Mantle test: r=0.895 indicates good fit of clustering

b) Dendrogram based on ISSR markers

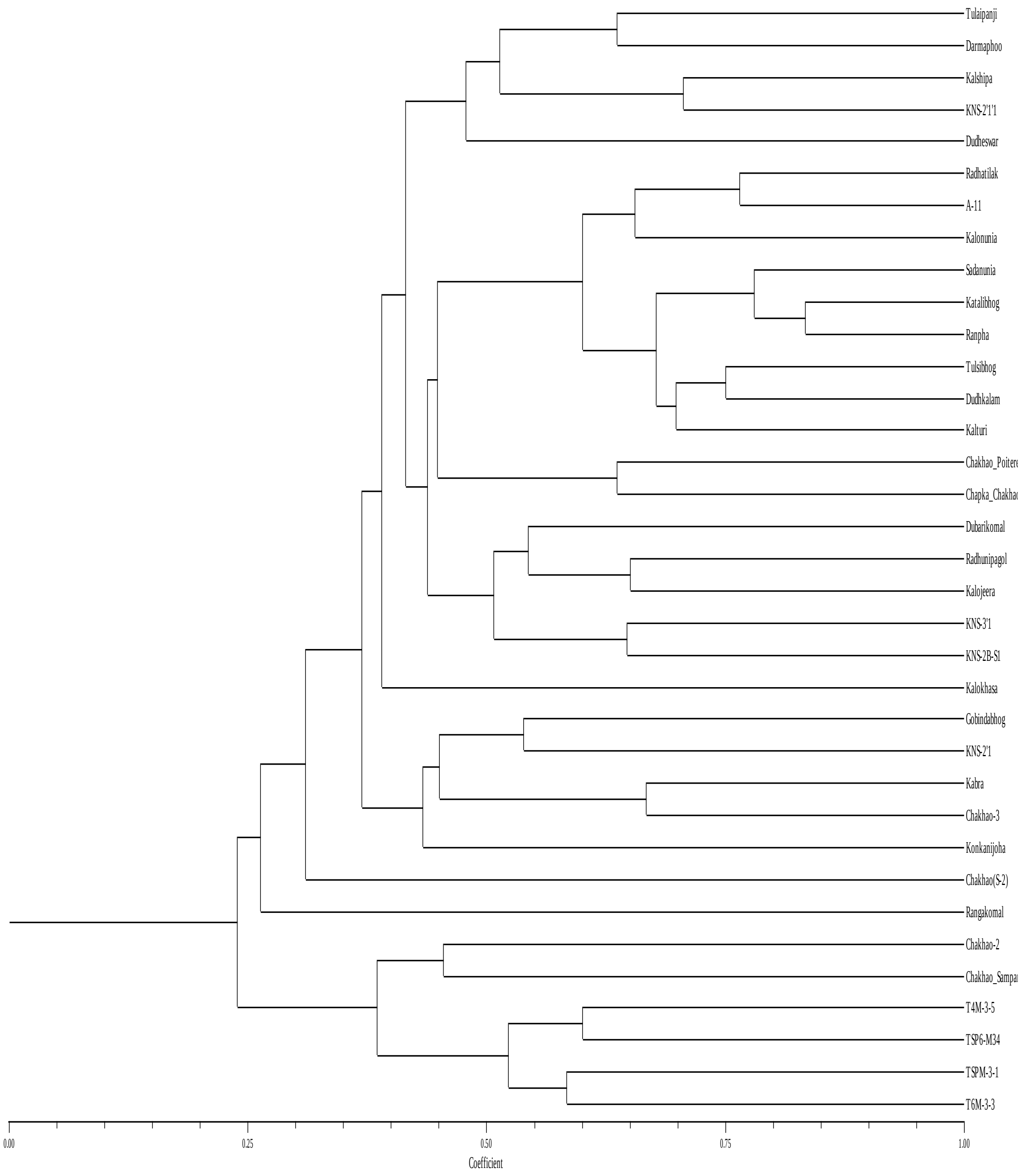

Mantle test: r=0.81 indicates good fit of clustering

Figure Legend: UPGMA based genetic clustering of thirty-five aromatic landraces determined by polymorphism obtained from SSR(a) and ISSR (b) markers using NTSYS-PC software version 1.80[30] (URL: http://www.exetersoftware.com/cat/ntsyspc/ntsyspc.html).

Supplementary Information 3: contribution of different parameters in PC in case of biochemical and disease related data. All the analysis was performed using R software[31], version 3.5.1, Patched (2018-07-02 r74950) Platform: x86_64-w64mingw32/x64 (64-bit) (https://www.R-project.org/).

a) **Biochemical Data**

	Eigen value	variance.percent	cumulative.variance.percent
Dim.1	2.176614	21.76614	21.76614
Dim.2	1.9740387	19.740387	41.50653
Dim.3	1.3836629	13.836629	55.34316
Dim.4	1.0861972	10.861972	66.20513
Dim.5	0.9644184	9.644184	75.84931
Dim.6	0.712849	7.12849	82.9778
Dim.7	0.6095667	6.095667	89.07347
Dim.8	0.5002169	5.002169	94.07564
Dim.9	0.4064769	4.064769	98.14041
Dim.10	0.1859592	1.859592	100

It has been observed that first PC is explaining 21.77% variation , second PC explaining 19.74% of variations, the third PC is explaining 13.84% variation and the fourth PC is explaining 10.86% of variations in the data. These four PCs together explaining 66.21% variation in the data. k-means non hierarchical cluster analysis was performed based on the PC results. As It has been observed that only four PC's are having eigen value more than 1, thus only three clusters are required. The optimal number of clusters were again verified using Gap statistic which again shows the same number of clusters to be made for the given data.

The contribution of the variables w.r.t. the first two PC's are as follows (which was plotted in color biplot diagram)

contribution		
	Dim.1	Dim.2
TSS	0.433043	24.341855
RES	12.634257	2.340947
NRS	19.239879	8.395359
STA	3.577254	4.105326
AMY	7.733857	5.039867
RS	18.833573	15.027366
GI	2.781554	28.380279
PRO	14.431764	2.237929
ANT	9.445757	6.660847
ARO	10.88906	3.470224

b) Phenotypic data

	Eigen value	variance.percent	cumulative.variance.percent
Dim.1	4.07324685	33.9437237	33.94372
Dim.2	2.75810253	22.9841878	56.92791
Dim.3	1.75669194	14.6390995	71.56701
Dim.4	0.93907339	7.8256116	79.39262
Dim.5	0.76415658	6.3679715	85.76059
Dim.6	0.53337495	4.4447913	90.20539
Dim.7	0.42915014	3.5762512	93.78164
Dim.8	0.25069108	2.0890924	95.87073
Dim.9	0.23935401	1.9946167	97.86535
Dim.10	0.13211866	1.1009888	98.96633
Dim.11	0.08480167	0.7066806	99.67302
Dim.12	0.03923819	0.3269849	100

It has been observed that first PC is explaining 33.94% variation , second PC explaining 22.98% of variations and the third PC is explaining 14.64% variation. These three PCs together explaining 71.57% variation in the data. k-means non hierarchical cluster analysis was performed based on the PC results. As It has been observed that only three PC's are having eigen value more than 1, thus only three clusters are required. The optimal number of clusters were again verified using Gap statistic which again shows the same number of clusters to be made for the given data.

The contribution of the variables w.r.t. the first two PC's are as follows

Variable	Dim.1	Dim.2
PLH	1.149087	7.481593
TIN	5.649553	5.9390921
LLA	6.127651	3.4152777
MLA	11.480949	1.6827179
ULA	14.37847	0.0060456
LLL	10.491176	9.2888641
LML	14.092739	8.1650378
LUL	14.829444	8.5366965
LST	2.264348	23.2623561
LSZ	2.877701	10.6599313
SPC	2.733046	18.8746612
AUD	13.925835	2.6877268

Supplementary Information 4: Correlation analysis between the Nutritional parameters and disease attributes. The correlation analysis between nutritional parameters and disease attributes were performed using SAS, version 9.3[32] (URL: https://www.sas.com/en_in/home.html).

The second value in each row indicates the p value. If the value is less than <.05, then the correlation is significant at 5% level of significance. If it is <.01, then it is significant at 1% level of significance. Correlation which are significant at 1%,

Pearson Correlation Coefficients, N = 70
Prob > |r| under H0: Rho=0

	Plh	Tin	Lla	Mla	Ula	Lll	Lml	Lul	Lst	Lss	Spc	AUD
**TSS**	0.23844<br>0.0468	-0.02649<br>0.8277	0.15300<br>0.2061	0.07186<br>0.5544	-0.08046<br>0.5079	-0.06325<br>0.6030	-0.06151<br>0.6130	-0.04340<br>0.7213	-0.08246<br>0.4974	0.05164<br>0.6712	0.08002<br>0.5102	0.10830<br>0.3722
**RES**	0.12526<br>0.3015	0.33421<br>0.0047	-0.26225<br>0.0283	-0.48394<br><.0001	-0.24662<br>0.0396	-0.10067<br>0.4070	-0.06494<br>0.5933	-0.05228<br>0.6673	-0.04869<br>0.6889	0.11202<br>0.3559	0.06782<br>0.5770	0.00605<br>0.9603
**NRS**	-0.18557<br>0.1240	-0.29838<br>0.0121	0.19830<br>0.0998	0.42483<br>0.0002	0.35968<br>0.0022	-0.14549<br>0.2295	-0.22433<br>0.0619	-0.20174<br>0.0940	0.35326<br>0.0027	0.23182<br>0.0535	0.37904<br>0.0012	-0.35851<br>0.0023
**STA**	0.06247<br>0.6074	-0.17531<br>0.1466	0.02972<br>0.8071	-0.00151<br>0.9901	0.09779<br>0.4206	-0.05082<br>0.6761	-0.01347<br>0.9119	-0.00254<br>0.9834	0.11249<br>0.3539	0.18735<br>0.1204	0.27935<br>0.0192	-0.12220<br>0.3136
**AMY**	-0.04749<br>0.6963	0.12243<br>0.3126	-0.02438<br>0.8412	-0.19267<br>0.1100	-0.21140<br>0.0790	-0.03473<br>0.7753	0.00357<br>0.9766	0.02609<br>0.8302	0.16256<br>0.1788	0.07084<br>0.5600	0.02658<br>0.8271	-0.08470<br>0.4857
**RS**	0.18247<br>0.1306	0.13475<br>0.2660	-0.17019<br>0.1590	-0.21176<br>0.0784	-0.20282<br>0.0922	-0.01759<br>0.8851	-0.02411<br>0.8430	0.01523<br>0.9004	-0.16951<br>0.1606	-0.22702<br>0.0588	-0.17425<br>0.1491	0.11765<br>0.3320
**GI**	-0.04606<br>0.7049	-0.04944<br>0.6844	0.06041<br>0.6193	0.09778<br>0.4206	-0.07463<br>0.5392	0.08617<br>0.4781	0.09254<br>0.4461	0.11917<br>0.3258	0.07380<br>0.5437	0.04756<br>0.6958	0.16464<br>0.1732	0.17685<br>0.1430
**PRO**	0.03147<br>0.7959	0.19075<br>0.1137	-0.11576<br>0.3399	-0.16108<br>0.1828	-0.10998<br>0.3648	-0.13252<br>0.2741	-0.12478<br>0.3034	-0.12713<br>0.2943	-0.17483<br>0.1477	-0.26073<br>0.0293	-0.14558<br>0.2292	-0.04862<br>0.6894
**ANT**	-0.22211<br>0.0646	-0.18893<br>0.1173	-0.14697<br>0.2247	-0.17592<br>0.1452	-0.18046<br>0.1349	0.07077<br>0.5605	0.11027<br>0.3635	0.16254<br>0.1788	0.31338<br>0.0083	-0.04403<br>0.7174	0.15780<br>0.1920	0.46666<br><.0001
**ARO**	-0.11208<br>0.3556	0.31736<br>0.0074	-0.01901<br>0.8759	-0.22979<br>0.0557	-0.28964<br>0.0150	0.03294<br>0.7866	0.08654<br>0.4763	0.10306<br>0.3959	-0.36791<br>0.0017	-0.29068<br>0.0146	-0.32653<br>0.0058	0.26511<br>0.0266

Supplementary Information 5: Distribution of pi genes in different genotypes

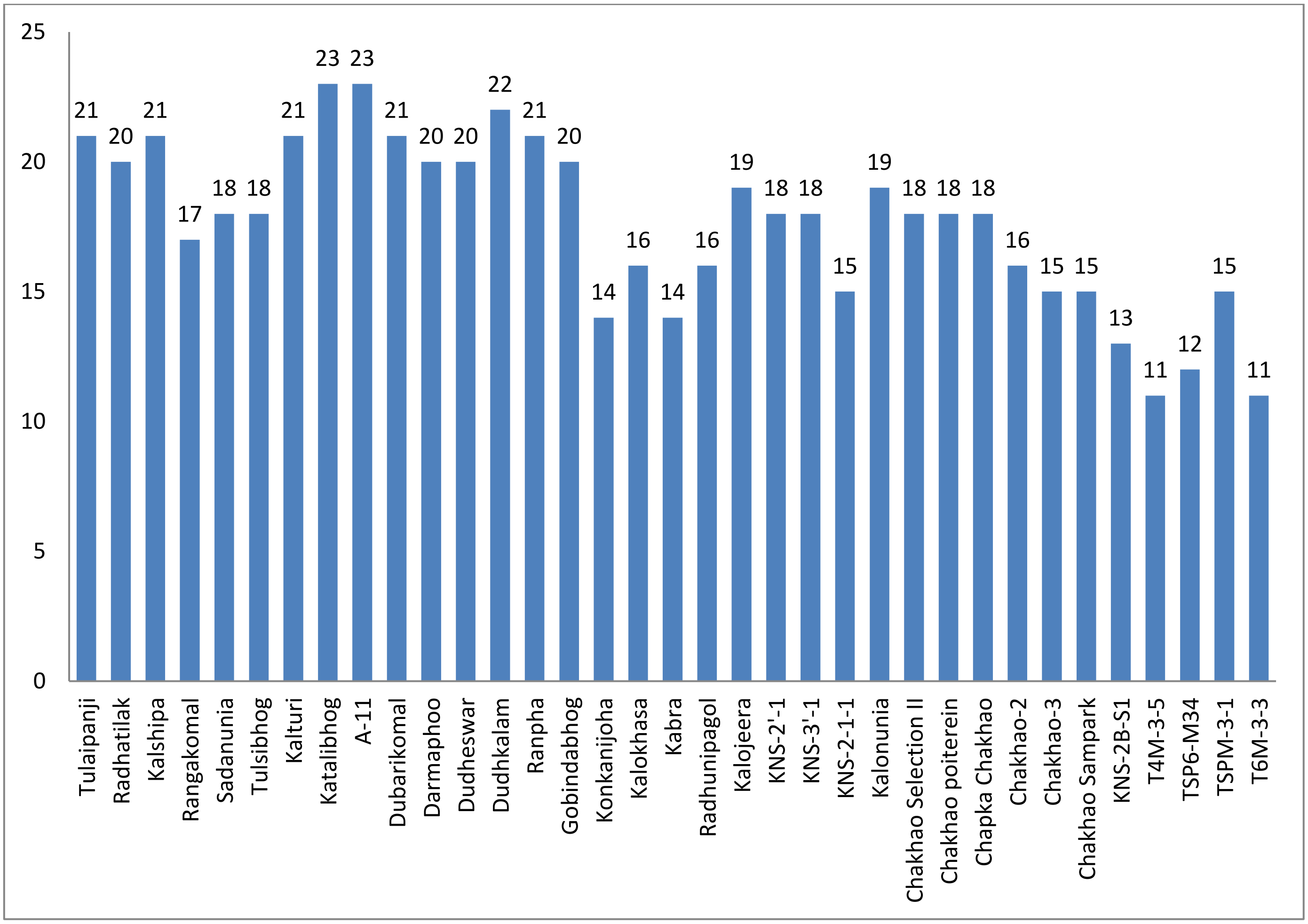

Supplementary Information 6: Kendall's tau-b measure of association with tolerance (AUDPC< 800) and susceptibility (AUDPC> 800) of the cultivars with presence and absence of *pi* genes tolerance and susceptibility are treated as 1 and 0 respectively. Kendall's tau-b correlation coefficient (τ_b) was calculated using IBM -*SPSS* (SPSS trial version 20[33], URL: https://www.ibm.com/analytics/spss-statistics-software).

Measure of association											
			Pid2	Pi36	Pi37	Pi5	Piz	Pizt	Pikp	Pikh	Pib
	AUDPC	Kendall's tau-b	.167	.168	.467	.	.101	.438	.459	.581	.
		Sig. (2-tailed)	.300	.300	.006	.	.400	.006	.006	.001	.
		N	35	35	35	35	35	35	35	35	35
**. Correlation is significant at the 0.01 level (2-tailed).											
*. Correlation is significant at the 0.05 level (2-tailed).											

Measure of association										
			Pi9	Pita_Pita2	Pik	Pi21	Pi22	Pi23	Pi1	Pikm
	AUDPC	Kendall's tau-b	.501	.512	.561	.112	-.122	-.070	-.056	.522
		Sig. (2-tailed)	..001	<.001	<.001	.060	.442	.599	.702	.193
		N	35	35	35	35	35	35	35	35
*. Correlation is significant at the 0.05 level (2-tailed).										
**. Correlation is significant at the 0.01 level (2-tailed).										

Measure of association									
			Pi61t	Pi2	Pik_duplicazte	Pi7t	Pizt_duplicate	Pi33	Pi27t
	AUDPC	Kendall's tau-b	.118	.024	.562	.055	.411	.411	.
		Sig. (2-tailed)	.069	.888	<.001	.569	.005	.006	.
		N	35	35	35	35	35	35	35
**. Correlation is significant at the 0.01 level (2-tailed).									
*. Correlation is significant at the 0.05 level (2-tailed).									

Supplementary information 7: The logistic regression model was fitted by considering only significant variables except AUDPC based on PCA results. Here, based on AUDPC values, disease occurrence has been calculated as 1 if AUDPC >1. Logistic regression modeling was carried out using IBM *Statistical Package for the Social Sciences*, (SPSS trial version 20[33], URL: https://www.ibm.com/analytics/spss-statistics-software).

The analysis suggests that LUL is highly significant at 1% level of significance whereas LML and ULA is significant at 5% level of significance. It has been observed that one unit increase in LUL will increase the odds in favor of blast occurrence by 2.439 times. On the other hand, one unit decrease of LML will increase the odds in favor of blast occurrence by 0.468 and one unit decrease in ULA will increase the odds in favor of blast occurrence by 0.720 times.

		B	S.E.	Sig.	Exp(B)
Step 1[a]	Lul	.892	.332	.007	2.439
	Lml	-.759	.463	.049	.468
	Lst	-2.845	1.467	.061	.058
	Lll	-.144	.421	.070	.866
	Spc	2.847	1.526	.057	17.244
	Ula	-.329	.149	.027	.720
	Constant	5.566	2.776	.045	261.457

The fitted model is:

$$P[Blast = 1] = \frac{1}{1 + \exp(5.566 + 0.892 * LUL - 0.759 * LML - 2.845 * LST - 0.144 * LLL + 2.847 * SPC - 0.329 * ULA)}$$

The **Hosmer and Lemeshow Test** is a goodness of fit of logistic model which is non significant (p vlaue 0.694) at 5% level of significance indicating that model fits well as for any goodness of fit ideally for a better fitting of the model the test should remain non significant.

Hosmer and Lemeshow Test

Step	Chi-square	df	Sig.
1	5.579	8	.694

It has been observed that, the fitted model is highly accurate in the sense that it is correctly able to classify presence or absence of blast disease based on the characters under consideration 75.7% times and it is even more accurate to correctly classify the presence of disease in the varieties under consideration 86.7% times.

Classification Table[a]

	Observed		Predicted		
			disease		Percentage Correct
			resistant or tolerant	susceptible	
Step 1	disease	resistant or tolerant	14	11	56.0
		susceptible	6	39	86.7
	Overall Percentage				75.7

a. The cut value is .500
